# Pooled analysis of WHO Surgical Safety Checklist use and mortality after emergency laparotomy

**DOI:** 10.1002/bjs.11051

**Published:** 2019-01-08

**Authors:** GlobalSurg Collaborative, Hannah S Thomas, Thomas G Weiser, Thomas M Drake, Stephen R Knight, Cameron Fairfield, Adesoji O Ademuyiwa, Maria Lorena Aguilera, Philip Alexander, Sara W Al‐Saqqa, Giuliano Borda‐Luque, Ainhoa Costas‐Chavarri, Faustin Ntirenganya, J Edward Fitzgerald, Stuart J Fergusson, James Glasbey, J.C. Allen Ingabire, Lawani Ismaïl, Hosni Khairy Salem, Anyomih Theophilus Teddy Kojo, Marie Carmela Lapitan, Richard Lilford, Andre L Mihaljevic, Dion Morton, Alphonse Zeta Mutabazi, Dmitri Nepogodiev, Adewale O Adisa, Riinu Ots, Francesco Pata, Thomas Pinkney, Tomas Poškus, Ahmad Uzair Qureshi, Antonio Ramos‐De la Medina, Sarah Rayne, Catherine A Shaw, Sebastian Shu, Richard Spence, Neil Smart, Stephen Tabiri, Aneel Bhangu, Ewen M Harrison, Ewen M Harrison, Azmina Verjee, Emmy Runigamugabo, Adesoji O Ademuyiwa, Adewale O Adisa, Maria Lorena Aguilera, Afnan Altamini, Philip Alexander, Sara W Al‐Saqqa, Giuliano Borda‐Luque, Jen Cornick, Ainhoa Costas‐Chavarri, Thomas M Drake, Stuart J Fergusson, J Edward Fitzgerald, James Glasbey, J.C. Allen Ingabire, Lawani Ismaïl, Zahra Jaffry, Hosni Khairy Salem, Chetan Khatri, Andrew Kirby, Anyomih Theophilus Teddy Kojo, Marie Carmela Lapitan, Richard Lilford, Andre L Mihaljevic, Midhun Mohan, Dion Morton, Alphonse Zeta Mutabazi, Dmitri Nepogodiev, Faustin Ntirenganya, Riinu Ots, Francesco Pata, Thomas Pinkney, Tomas Poškus, Ahmad Uzair Qureshi, Antonio Ramos‐De la Medina, Sarah Rayne, Gustavo Recinos, Kjetil Søreide, Catherine A Shaw, Sebastian Shu, Richard Spence, Neil Smart, Stephen Tabiri, Ewen M Harrison, Aneel Bhangu, Chetan Khatri, Neel Gobin, Ana Vega Freitas, Nigel Hall, Sung‐Hee Kim, Ahmed Negida, Hosni Khairy, Zahra Jaffry, Stephen J Chapman, Alexis P Arnaud, Stephen Tabiri, Gustavo Recinos, Cutting Edge Manipal, Midhun Mohan, Radhian Amandito, Marwan Shawki, Michael Hanrahan, Francesco Pata, Justas Zilinskas, April Camilla Roslani, Cheng Chun Goh, Adesoji O Ademuyiwa, Gareth Irwin, Sebastian Shu, Laura Luque, Hunain Shiwani, Afnan Altamimi, Mohammed Ubaid Alsaggaf, Stuart J Fergusson, Richard Spence, Sarah Rayne, Jenifa Jeyakumar, Yucel Cengiz, Dmitri A Raptis, James C Glasbey, Maria Marta Modolo, Dushyant Iyer, Sebastian King, Tom Arthur, Sayeda Nazmum Nahar, Ade Waterman, Lawani Ismaïl Walsh, Arnav Agarwal, Augusto Zani, Mohammed Firdouse, Tyler Rouse, Qinyang Liu, Juan Camilo Correa, Hosni Khairy Salem, Peep Talving, Mengistu Worku, Alexis Arnaud, Stephen Tabiri, Vassilis Kalles, Maria Lorena Aguilera, Gustavo Recinos, Basant Kumar, Sunil Kumar, Radhian Amandito, Roy Quek, Francesco Pata, Luca Ansaloni, Ahmed Altibi, Donatas Venskutonis, Justas Zilinskas, Tomas Poskus, John Whitaker, Vanessa Msosa, Yong Yong Tew, Alexia Farrugia, Elaine Borg, Antonio Ramos‐De la Medina, Zineb Bentounsi, Adesoji O Ademuyiwa, Kjetil Søreide, Tanzeela Gala, Ibrahim Al‐Slaibi, Haya Tahboub, Osaid H. Alser, Diego Romani, Sebestian Shu, Piotr Major, Aurel Mironescu, Matei Bratu, Amar Kourdouli, Aliyu Ndajiwo, Abdulaziz Altwijri, Mohammed Ubaid Alsaggaf, Ahmad Gudal, Al Faifi Jubran, Sam Seisay, Bettina Lieske, Sarah Rayne, Richard Spence, Irene Ortega, Jenifa Jeyakumar, Kithsiri J. Senanayake, Omar Abdulbagi, Yucel Cengiz, Dmitri Raptis, Yuksel Altinel, Chia Kong, Ella Teasdale, Gareth Irwin, Michael Stoddart, Rakan Kabariti, Sukrit Suresh, Katherine Gash, Ragavan Narayanan, Mayaba Maimbo, Claudio Fermani, Ruben Balmaceda, Maria Marta Modolo, Ewan Macdermid, Neel Gobin, Roxanne Chenn, Cheryl Ou Yong, Michael Edye, Martin Jarmin, Scott K D'amours, Dushyant Iyer, Daniel Youssef, Nicholas Phillips, Jason Brown, Robert George, Cherry Koh, Oliver Warren, Isaac Hanley, Marilla Dickfos, Clemens Nawara, Dietmar Öfner, Florian Primavesi, Ashrarur Rahman Mitul, Khalid Mahmud, Margub Hussain, Hafiz Hakim, Tapan Kumar, Antje Oosterkamp, Pamphile A Assouto, Ismail Lawani, Yacoubou Imorou Souaibou, Aung Kyaw Tun, Chean Leung Chong, Giridhar H Devadasar, Chean Leung Chong, Muhammad Rashid Minhas Qadir, Kyaw Phyo Aung, Lee Shi Yeo, Chean Leung Chong, Vanessa Dina Palomino Castillo, Monique Moron Munhoz, Gisele Moreira, Luiz Carlos Barros De Castro Segundo, Salim Anderson Khouri Ferreira, Maíra Cassa Careta, Stella Binna Kim, Alexandre Venancio De Sousa, Alyne Daltri Lazzarini Cury, Gustavo Peixoto Soares Miguel, Ana Vega Carreiro De Freitas, Barbara Pereira Silvestre, Julia Guasti Pinto Vianna, Carolina Oliveira Felipe, Luis Alberto Valente Laufer, Fernanda Altoe, Luana Ayres Da Silva, Marina Luiza Pimenta, Thiago Fernandes Giuriato, Paulo Alves Bezerra Morais, Jessica Souza Luiz, Rafael Araujo, Juliana Menegussi, Marisa Leal, Caio Vinícius Barroso de Lima, Luiza Sarmento Tatagiba, Antônio Leal, Diogo Vinicius dos Santos, Gustavo Pereira Fraga, Romeo Lages Simoes, Simon Stock, Samuel Nigo, Juana Kabba, Tagang Ebogo Ngwa, James Brown, Sebastian King, Augusto Zani, Georges Azzie, Mohammed Firdouse, Sameer Kushwaha, Arnav Agarwal, Karen Bailey, Brian Cameron, Michael Livingston, Alexandre Horobjowsky, Dan L Deckelbaum, Tarek Razek, Boris Marinkovic, Eugenio Grasset, Nicole D'aguzan, Eugenio Grasset, Julio Jimenez, Roberto Macchiavello, Zhongtao Zhang, Wei Guo, Junyeong Oh, Fei Zheng, Irene Montes, Sebastian Sierra, Manuela Mendez, Maria Isabel Villegas, Maria Clara Mendoza Arango, Ivan Mendoza, Fred Alexander Naranjo Aristizã¡bal, Jaime Andres Montoya Botero, Victor Manuel Quintero Riaza, Jakeline Restrepo, Carlos Morales, Maria Clara Mendoza Arango, Herman Cruz, Alejandro Munera, Maria Clara Mendoza Arango, Robert Karlo, Edgar Domini, Jakov Mihanovic, Mihael Radic, Kresimir Zamarin, Nikica Pezelj, Manuel Hache‐Marliere, Sylvia Batista Lemaire, Ruben Rivas, Ahmed Khyrallh, Ahamed Hassan, Gamal Shimy, Mohamed A Baky Fahmy, Ayman Nabawi, Mohamed Elfil, Mohamed Ghoneem, Muhammad El‐Saied Ahmad Muhammad Gohar, Mohamed Asal, Mostafa Abdelkader, Mahmoud Gomah, Hayssam Rashwan, Mohamed Karkeet, Ahmed Gomaa, Amr Hasan, Ahmed Elgebaly, Omar Saleh, Ahmad Abdel Fattah, Abdullah Gouda, Abd Elrahman Elshafay, Abdalla Gharib, Ahmed Menshawy, Mohammed Hanafy, Abdullah Al‐Mallah, Mahmoud Abdulgawad, Mohamad Baheeg, Mohammed Alhendy, Ibrahim AbdelFattah, Abdalla Kenibar, Omar Osman, Mostafa Gemeah, Ahmed Mohammed, Abdalrahman Adel, Abdalla Gharib, Abdelrahman Mohammed, Abdelrahman Sayed, Mohamed Abozaid, Ahmed Hafez El‐Badri Kotb, Ali Amin Ahmed Ata, Mohammed Nasr, Abdelrahman Alkammash, Mohammed Saeed, Nader Abd El Hamid, Attia Mohamed Attia, Ahmed Abd El Galeel, Eslam Elbanby, Khalid Salah El‐Dien, Usama Hantour, Omar Alahmady, Billal Mansour, Amr Muhammad Elkorashy, Emad Mohamed Saeed Taha, Kholod Tarek Lasheen, Salma Said Elkolaly, Nehal Yosri Elsayed Abdel‐Wahab, Mahmoud Ahmed Fathi Abozyed, Ahmed Adel, Ahmed Moustafa Saeed, Gehad Samir El Sayed, Jehad Hassan Youssif, Soliman Magdy Ahmed, Nermeen Soubhy El‐Shahat, Abd El‐Rahman Hegazy Khedr, Abdelrhman Osama Elsebaaye, Mohamed Elzayat, Mohamed Abdelraheim, Ibrahim Elzayat, Mahmoud Warda, Khaled Naser El Deen, Abdelrhman Essam Elnemr, Omar Salah, Mohamed Abbas, Mona Rashad, Ibrahim Elzayyat, Dalia Hemeda, Gehad Tawfik, Mai Salama, Hazem Khaled, Mohamed Seisa, Kareem Elshaer, Abdelfatah Hussein, Mahmoud Elkhadrawi, Ahmed Mohamed Afifi, Osama Saadeldeen Ebrahim, Mahmoud Mohamed Metwally, Rowida Elmelegy, Diaa Moustafa Elbendary Elsawahly, Hisham Safa, Eman Nofal, Mohamed Elbermawy, Ahmed Abdelmotaleb Ghazy, Hisham Samih, Asmaa Abdelgelil, Sarah Abdelghany, Ahmed El Kholy, Metwally Aboraya, Fatma Elkady, Mahmoud Salma, Sarah Samy, Reem Fakher, Aya Aboarab, Ahmed Samir, Ahmed Sakr, Abdelrahman Haroun, Asmaa Abdel‐Rahman Al‐Aarag, Ahmed Elkholy, Sally Elshanwany, Esraa Ghanem, Ahmed Tammam, Ali Mohamed Hammad, Yousra El Shoura, Gehad El Ashal, Hosni Khairy, Sarah Antar, Sara Mehrez, Mahmoud Abdelshafy, Maha Gamal Mohamad Hamad, Mona Hosh, Emad Abdallah, Basma Magdy, Thuraya Alzayat, Elsayed Gamaly, Hossam Elfeki, Amany Abouzahra, Shereen Elsheikh, Fatimah I Elgendy, Fathia Abd El‐Salam, Osama Seifelnasr, Mohamed Ammar, Athar Eysa, Aliaa Sadek, Aliaa Gamal Toeema, Aly Nasr, Mohamed Abuseif, Hagar Zidan, Sara Abd Elmageed Barakat, Nadin Elsayed, Yasmin Abd Elrasoul, Ahmed Elkelany, Mohamed Sabry Ammar, Mennat‐Allah Mustafa, Yasmin Hegazy, Mohamed Etman, Samar Saad, Mahmoud Alrahawy, Ahmed Raslan, Mahmoud Morsi, Ahmed Rslan, Ahmed Sabry, Hager Elwakil, Heba Shaker, Hagar Zidan, Yasmin Abd‐Elrasoul, Ahmed Elkelany, Hussein El‐Kashef, Mohamed Shaalan, Areej Tarek, Ayman Elwan, Ahmed Ragab Nayel, Mostafa Seif, Ayman Elwan, Doaa Emadeldin, Mohamed Ali Ghonaim, Ahmad Almallah, Ahmed Fouad, Eman Adel Sayma, Ahmad Elbatahgy, Angham Solaiman El‐Ma'doul, Ahmed Mosad, Hager Tolba, Diaa Eldin Abdelazeem Amin Elsorogy, Hassan Ali Mostafa, Amira Atef Omar, Ola Sherief Abd El Hameed, Ahmed Lasheen, Yasser Abd El Salam, Ashraf Morsi, Mohammed Ismail, Hager Ahmed El‐badawy, Mohamed A Amer, Ahmed Elkelany, Ahmed Elkelany, Ahmed Sabry El‐Hamouly, Noura A. Attallah, Omnia Mosalum, Ahmed Afandy, Ahmed Mokhtar, Alaa Abouelnasr, Sara Ayad, Ramdan Shaker, Rokia Sakr, Ramadan Shaker, Mahmoud Amreia, Soaad Elsobky, Mohamed Mustafa, Ahmed Abo El Magd, Abeer Marey, Amr Tarek Hafez, Mohamed F Zalabia, Mohamed Moamen Mohamed, Amr Fadel, Emad Ali Ahmed, Ahmad Ali, Mohammad Ghassan Alwafai, Abdullah Dwydar, Sara Kharsa, Ehab Mamdouh, Hatem El‐Sheemy, Ibrahim AlYoussef, Abouelatta Khairy Aly, Ahmad Aldalaq, Ehab Alnawam, Dalia Alkhabbaz, Mahmoud Saad, Shady Hussein, Ahmed Abo Elazayem, Ahmed Meshref, Marwa Elashmawy, Mohammed Mousa, Ahmad Nashaat, Sara Ghanem, Zaynab M Elsayed, Aya Elwaey, Iman Elkadsh, Mariam Darweesh, Ahmed Mohameden, Mennaallah Hafez, Ahmed Badr, Assmaa Badwy, Mohamed Abd El Slam, Mohamed Elazoul, Safwat Al‐Nahrawi, Lotfy Eldamaty, Fathee Nada, Mohamed Ameen, Aya Hagar, Mohamed Elsehimy, Mohammad Aboraya, Hossam Dawoud, Shorouk El Mesery, Abeer El Gendy, Ahmed Abdelkareem, Ahmed Safwan Marey, Mostafa Allam, Sherif Shehata, Khaled Abozeid, Marwa Elshobary, Ahmed Fahiem, Sameh Sarsik, Amel Hashish, Mohamed Zidan, Mohamed Hashish, Shaimaa Aql, Abdelaziz Osman Abdelaziz Elhendawy, Mohamed Husseini, Esraa Kasem, Ahmed Gheith, Yasmin Elfouly, Ahmed Ragab Soliman, Yasmein Ibrahim, Nesma Elfouly, Ahmed Fawzy, Ahmed Hassan, Mohammad Rashid, Abdallah Salah Elsherbiny, Basem Sieda, Nermin M Badwi, Mohammed Mustafa Hassan Mohammed, Osama Mohamed, Mohammad Abdulkhalek Habeeb, Mengistu Worku, Nichole Starr, Semay Desta, Sahlu Wondimu, Nebyou Seyoum Abebe, Efeson Thomas, Frehun Ayele Asele, Daniel Dabessa, Nebiyou Seyoum Abebe, Abebe Bekele Zerihun, Panu Mentula, Ari Leppäniemi, Ville Sallinen, Aurelien Scalabre, Fernanda Frade, Sabine Irtan, Vivien Graffeille, Elodie Gaignard, Quentin Alimi, Quentin Alimi, Vivien Graffieille, Elodie Gaignard, Olivier Abbo, Sofia Mouttalib, Ourdia Bouali, Erik Hervieux, Yves Aigrain, Nathalie Botto, Alice Faure, Lucile Fievet, Nicoleta Panait, Emilie Eyssartier, Francoise Schmitt, Guillaume Podevin, Valentine Parent, Amandine Martin, Alexis Pierre Arnaud, Cecile Muller, Arnaud Bonnard, Matthieu Peycelon, Francis Abantanga, Kwaku Boakye‐Yiadom, Mohammed Bukari, Frank Owusu, Joseph Awuku‐Asabre, Stephen Tabiri, Lemuel Davies Bray, Dimitrios Lytras, Kyriakos Psarianos, Anastasia Bamicha, Eirini Kefalidi, Georgios Gemenetzis, Christos Dervenis, Nikolaos Gouvas, Christos Agalianos, Michail Kontos, Gregory Kouraklis, Dimitrios Karousos, Stylianos Germanos, Constantinos Marinos, Christos Anthoulakis, Nikolaos Nikoloudis, Nikolaos Mitroudis, Gustavo Recinos, Sergio Estupinian, Walter Forno, José René Arévalo Azmitia, Carla Cecilia Ramãrez Cabrera, Romeo Guevara, Maria Aguilera, Napoleon Mendez, Cesar Augusto Azmitia Mendizabal, Pablo Ramazzini, Mario Contreras Urquizu, Fernando Tale, Rafael Soley, Emanuel Barrios, Emmanuel Barrios, Daniel Estuardo Marroquín Rodríguez, Carlos Iván Pérez Velásquez, Sara María Contreras Mérida, Francisco Regalado, Mario Lopez, Miguel Siguantay, Fong Yee Lam, Kylie Joan‐yi Szeto, Charing Cheuk Ling Szeto, Wing Sum Li, Kieran Ka Kei Li, Man Fung Leung, Tony Mak, Simon Ng, SS Prasad, Anand Kirishnan, Nidhi Gyanchandani, Bylapudi Seshu Kumar, Muthukumaran Rangarajan, Sriram Bhat, Anjana Sreedharan, S.V. Kinnera, Yella Reddy, Caranj Venugopal, Sunil Kumar, Abhishek Mittal, Shravan Nadkarni, Harish Neelamraju Lakshmi, Puneet Malik, Neel Limaye, Srinivas Pai, Pratik Jain, Monty Khajanchi, Savni Satoskar, Rajeev Satoskar, Abid Bin Mahamood, Eldaa Prisca Refianti Sutanto, Daniel Ardian Soeselo, Chintya Tedjaatmadja, Fitriana Nur Rahmawati, Radhian Amandito, Maria Mayasari, Ruqaya Kadhim Mohammed Jawad Al‐Hasani, Hasan Ismael Ibraheem Al‐Hameedi, Hasan Ismael Ibraheem, Israa Abdullah Aziz Al‐Azraqi, Lubna Sabeeh, Rahma Kamil, Marwan Shawki, Muwaffaq Mezeil Telfah, Amoudtha Rasendran, Jacqueline Sheehan, Robert Kerley, Caoimhe Normile, Richard William Gilbert, Jiheon Song, Mohamed Dablouk, Linnea Mauro, Mohammed Osman Dablouk, Michael Hanrahan, Paul Kielty, Eleanor Marks, Simon Gosling, Michelle Mccarthy, Amoudtha Rasendran, Diya Mirghani, Syed Altaf Naqvi, Chee Siong Wong, Siyi Chung, Reuban D'cruz, Ronan Cahill, Simon George Gosling, Michelle Mccarthy, Amoudtha Rasendran, Ciara Fahy, Jiheon Song, Michael Hanrahan, Diana Duarte Cadogan, Anna Powell, Richard Gilbert, Caroline Clifford, Caoimhe Normile, Aoife Driscoll, Stassen Paul, Chris Lee, Ross Bowe, William Hutch, Michael Hanrahan, Helen Mohan, Maeve O'neill, Kenneth Mealy, Piergiorgio Danelli, Andrea Bondurri, Anna Maffioli, Mario Pasini, Giacomo Pata, Stefano Roncali, Paolo Silvani, Michele Carlucci, Roberto Faccincani, Luigi Bonavina, Yuri Macchitella, Chiara Ceriani, Gregorio Tugnoli, Salomone Di Saverio, Khaled Khattab, Miguel Angel Paludi, Domenica Pata, Luigi Maria Cloro, Andrea Allegri, Luca Ansaloni, Federico Coccolini, Ezio Veronese, Luca Bortolasi, Alireza Hasheminia, Giacomo Nastri, Massimiliano Dal Canto, Stefano Cucumazzo, Francesco Pata, Angelo Benevento, Gaetano Tessera, Pier Paolo Grandinetti, Alessio Maniscalco, Giovanni Luca Lamanna, Luca Turati, Giovanni Sgroi, Emanuele Rausa, Roberta Villa, Michela Monteleone, David Merlini, Federico Coccolini, Luca Ansaloni, Andrea Allegri, Veronica Grassi, Roberto Cirocchi, Alban Cacurri, Hamza Waleed, Ahmed Diab, Fathi Elzowawi, Mantas Jokubauskas, Karolis Varkalys, Donatas Venskutonis, Robertas Pranevicius, Viktorija Ambrozeviciute, Simona Juciute, Austė Skardžiukaitė, Donatas Venskutonis, Saulius Bradulskis, Linas Urbanavicius, Aiste Austraite, Romualdas Riauka, Justas Zilinskas, Zilvinas Dambrauskas, Paulius Karumnas, Zigmantas Urniezius, Reda Zilinskiene, Anele Rudzenskaite, Ausrine Usaityte, Margarita Montrimaite, Nerijus Kaselis, Andrius Strazdas, Kristijonas Jokubonis, Kornelija Maceviciute, Virgilijus Beisa, Tomas Poskus, Kestutis Strupas, Erikas Laugzemys, Andrej Kolosov, Valdemaras Jotautas, Ignas Rakita, Saulius Mikalauskas, Darius Kazanavicius, Rokas Rackauskas, Kestutis Strupas, Tomas Poskus, Virgilijus Beisa, Ritauras Rakauskas, Egle Preckailaite, Ross Coomber, Kenneth Johnson, Jennifer Nowers, Dineshwary Periasammy, Afizah Salleh, Andre Das, Reuben Goh Ern Tze, Milaksh Nirumal Kumar, Nik Azim Nik Abdullah, Nik Ritza Kosai, Mustafa Taher, Reynu Rajan, Hoong Yin Chong, April Camilla Roslani, Cheng Chun Goh, Marija Agius, Elaine Borg, Maureen Bezzina, Roberta Bugeja, Martinique Vella‐Baldacchino, Andrew Spina, Josephine Psaila, Helene Francois‐Coridon, Cecilia Tolg, Jean‐Francois Colombani, Diaz‐Zorrilla, Antonio Ramos‐ De La Medina, Samantha Corro‐Diaz Gonzalez, Mário Jacobe, Domingos Mapasse, Elizabeth Snyder, Ramadan Oumer, Mohammed Osman, Aminu Mohammad, Lofty‐John Anyanwu, Abdulrahman Sheshe, Alaba Adesina, Olubukola Faturoti, Ogechukwu Taiwo, Muhammad Habib Ibrahim, Abdulrasheed A Nasir, Siyaka Itopa Suleiman, Adewale Adeniyi, Opeoluwa Adesanya, Ademola Adebanjo, Roland Osuoji, Kazeem Atobatele, Ayokunle Ogunyemi, Omolara Williams, Mobolaji Oludara, Olabode Oshodi, Adesoji Ademuyiwa, AbdulRazzaq Oluwagbemiga Lawal, Felix Alakaloko, Olumide Elebute, Adedapo Osinowo, Christopher Bode, Abidemi Adesuyi, Adesoji Tade, Adeleke Adekoya, Collins Nwokoro, Omobolaji O Ayandipo, Taiwo Akeem Lawal, Akinlabi E Ajao, Samuel Sani Ali, Babatunde Odeyemi, Samson Olori, Ademola Popoola, Ademola Adeyeye, James Adeniran, William J. Lossius, Ingemar Havemann, Kenneth Thorsen, Jon Kristian Narvestad, Kjetil Soreide, Trude Beate Wold, Linn Nymo, Mohammed Elsiddig, Manzoor Dar, Kamran Faisal Bhopal, Zainab Iftikhar, Muhammad Mohsin Furqan, Bakhtiar Nighat, Masood Jawaid, Abdul Khalique, Ahsan Zil‐E‐Ali, Anam Rashid, Hasnain Abbas Dharamshi, Tahira Naqvi, Ahmad Faraz, Abdul Wahid Anwar, Tahir Muhammad Yaseen, Ghina Shamim Shamsi, Ghina Shamsi, Tahir Yaseen, Wahid Anwer, Horacio Paredes Decoud, Omar Aguilera, Ismael Isaac Zelada Alvarez, Juan Marcelo Delgado, Gustavo Miguel Machain Vega, Helmut Alfredo Segovia Lohse, Wendy Leslie Messa Aguilar, Jose Antonio Cabala Chiong, Ana Cecilia Manchego Bautista, Eduardo Huaman, Sergio Zegarra, Rony Camacho, Jose María Vergara Celis, Diego Alonso Romani Pozo, José Hamasaki, Edilberto Temoche, Jaime Herrera‐Matta, Carla Pierina García Torres, Luis Miguel Alvarez Barreda, Ronald Renato Barrionuevo Ojeda, Octavio Garaycochea, Melanie Castro Mollo, Mitchelle Solange De Fã Tima Linares Delgado, Francisco Fujii, Ana Cecilia Manchego Bautista, Wendy Leslie Messa Aguilar, Jose Antonio Cabala Chiong, Susana Yrma Aranzabal Durand, Carlos Alejandro Arroyo Basto, Nelson Manuel Urbina Rojas, Sebastian Bernardo Shu Yip, Ana Lucia Contreras Vergara, Andrea Echevarria Rosas Moran, Giuliano Borda Luque, Manuel Rodriguez Castro, Ramon Alvarado Jaramillo, George Manrique Sila, Crislee Elizabeth Lopez, Mardelangel Zapata Ponze De Leon, Massiell Machaca, Ronald Coasaca Huaraya, Andy Arenas, Crislee López, Clara Milagros Herrera Puma, Wilfredo Pino, Christian Hinojosa, Melanie Zapata Ponze De Leon, Susan Limache, George Manrrique Sila, Layza‐Alejandra Mercado Rodriguez, Renato Melo, Jose Costa‐Maia, Nuno Muralha, Frederique Sauvat, Ionasc Dan, Mircea Hogea, Pandi Eduard, Razvan‐Matei Bratu, Mircea Beuran, Ionut‐Bogdan Diaconescu, Bogdan‐Valeriu Martian, Florin‐Mihail Iordache, Mihaela Vartic, Lucian Corneliu Vida, Liviu Iuliu Muntean, Aurel Sandu Mironescu, Vizir Jean Paul Nsengimana, Alice Niragire, Jean De La Croix Allen Ingabire, Eugene Niyirera, Nicola Zanini, Elio Jovine, Giovanni Landolfo, Ibrahim N. Alomar, Saleh A. Alnuqaydan, Abdulrahman M. Altwigry, Moayad Othman, Nohad Osman, Enas Alqahtani, Mohammed Alzahrani, Rifan Alyami, Emad Aljohani, Ibrahim Alhabli, Zaher Mikwar, Sultan Almuallem, Emad Aljohani, Rifan Alyami, Mohammed Alzahrani, Abrar Nawawi, Mohamad Bakhaidar, Ashraf A. Maghrabi, Mohammed Alsaggaf, Murad Aljiffry, Abdulmalik Altaf, Ahmad Khoja, Alaa Habeebullah, Nouf Akeel, Nashat Ghandora, Abdullah Almoflihi, Abdulmalik Huwait, Abeer Al‐shammari, Mashael Al‐Mousa, Masood Alghamdi, Walid Adham, Bandar Albeladi, Muayad Ahmed Alfarsi, Atif Mahdi, Saad Al Awwad, Afnan Altamimi, Thamer Nouh, Mazen Hassanain, Salman Aldhafeeri, Nawal Sadig, Osama Algohary, Mohannad Aledrisy, Ahmad Gudal, Ahmad Alrifaie, Mohammed AlRowais, Amani Althwainy, Alaa Shabkah, Uthman Alamoudi, Mawaddah Alrajraji, Basim Alghamdi, Saud Aljohani, Abdullah Daqeeq, Jubran J Al‐Faifi, Vicky Jennings, Nyawira Ngayu, Rachel Moore, Victor Kong, Hayden Kretzmann, Katie Connor, Daniel Nel, Colleen Sampson, Richard Spence, Eugenio Panieri, Sarah Rayne, Nosisa Sishuba, Myint Tun, Albert Mohale Mphatsoe, Jo‐Anne Carreira, Ella Teasdale, Mark Wagener, Stefan Botes, Danelo Du Plessis, Fernando Fernandez‐Bueno, Jose Aguilar‐Jimenez, Jose Andres Garcia‐Marin, Lorena Solar García, Luis Joaquín García Florez, Rubén Darío Arias Pacheco, Janet Pagnozzi, Jimy Harold Jara Quezada, Jose Luis Rodicio, German Minguez, Raquel Rodríguez‐Uría, Paul Ugalde, Camilo Lopez‐Arevalo, Luis Barneo, Jessica Patricia Gonzales Stuva, Irene Ortega‐Vazquez, Lorena Rodriguez, Norberto Herrera, Prasad Pitigala Arachchi, Wanigasekara Senanayake Mudiyanselage Kithsiri Janakantha Senanayake, Lalith Asanka Jayasooriya Jayasooriya Arachchige, Sivasuriya Sivaganesh, Dulan Irusha Samaraweera, Vimalakanthan Thanusan, Ahmed Elgaili Khalid Musa, Reem Mohammed Hassan Balila, Mohamed Awad Elkarim Hamad Mohamed, Hussein Ali, Hagir Zain Elabdin, Alaa Hassan, Sefeldin Mahdi, Hala Ahmed, Sahar Abdoun Ishag Idris, Makki Elsayed, Mohammed Elsayed, Mohamed Mahmoud, Magnus Boijsen, Per‐Olof Lundgren, Ulf Gustafsson, Ali Kiasat, Fredrik Wogensen, Fredrik Wogensen, Emma Jurdell, Anders Thorell, Hildur Thorarinsdottir, Maria Utter, Sami Martin Sundstrom, Cecilia Wredberg, Ann Kjellin, Johanna Nyberg, Bjorn Frisk, Malin Sund, Linda Andersson, Ulf Gunnarsson, Yücel Cengiz, Sandra Ahlqvist, Ida Björklund Hanna Royson, Per Weber, Hans‐Ivar Pahlsson, Eva Borin, Maria Hjertberg, Hanna Royson, Per Weber, Roger Schmid, Debora Schivo, Vasileios Despotidis, Stefan Breitenstein, Ralph F Staerkle, Erik Schadde, Fabian Deichsel, Alexandra Gerosa, Antonio Nocito, Dimitri Aristotle Raptis, Barbara Mijuskovic, Markus Zuber, Lukas Eisner, Swantje Kruspi, Katharina Beate Reinisch, Christin Schoewe, Allan Novak, Adrian F. Palma, Gerfried Teufelberger, Msafiri Kimaro, Rachel King, Ali Zeynel Abidin Balkan, Mehmet Gumar, Mehmet Ali Yavuz, Ufuk Karabacak, Gokhan Lap, Bahar Busra Ozkan, Bahar Busra Ozkan, Murat Karakahya, Ryan Adams, Robert Morton, Liam Henderson, Ruth Gratton, Keiran David Clement, Kate Yu‐Ching Chang, David Mcnish, Ryan Mcintosh, William Milligan, Brendan Skelly, Hannah Anderson‐Knight, Roger Lawther, Jemina Onimowo, Veereanna Shatkar, Shivanee Tharmalingam, Evelina Woin, Tessa Fautz, Oliver Ziff, Shiva Dindyal, Sam Arman, Shagorika Talukder, Sam Arman, Vijay Gadhvi, Shagorika Talukder, Luen Shaun Chew, Jonathan Heath, Natalie Blencowe, Sally Hallam, Katherine Gash, Gurdeep Singh Mannu, Dimitris‐Christos Zachariades, Ailsa Claire Snaith, Thusitha Sampath Hettiarachchi, Arjun Nesaratnam, James Wheeler, Darragh McCullagh, Joshua Michael Clements, Ata Khan, Foteini Koumpa, Christina Neophytou, Jessica Roth, Wai Cheong Soon, Mohammed Deputy, Ahmed Ahmed, Annelisse Ashton, Joe Vincent, Jack Almy, Taufiq Khan, John Lee Y Allen, Charlotte Jane Mcintyre, Dominic Charles Marshall, Mark Sykes, Nebil Behar, Harriet Jordan, Yaseen Rajjoub, Thomas Sherman, Timothy White, Anna Watts, Rohan Ardley, Tan Arulampalam, Apar Shah, Damien Brown, Emma Blower, Paul Sutton, Konstantinos Gasteratos, Dale Vimalachandran, Cathy Magee, Gareth Irwin, Andrew Mcguigan, Stephen Mcaleer, Clare Morgan, Sarah Braungart, Kirsten Lafferty, Peter Labib, Andrei Tanase, Clodagh Mangan, Lillian Reza, Lillian Reza, Andrei Tanase, Clodagh Mangan, Helen Woodward, Craig Gouldthorpe, Megan Turner, Jonathan R L Wild, Tom AM Malik, Victoria K Proctor, Kalon Hewage, James Davies, Andre Dubois, Sayed Sarwary, Ali Zardab, Alan Grant, Robert Mcintyre, Yogendra Praveen Mogan, Weiguang Ho, Bryon Frankie Hon Khi Chong, Shirish Tewari, Gemma Humm, Eriberto Farinella Nigel J Hall, Naomi J Wright, Christina P Major, Thelma Xerri, Phoebe De Bono, Jasim Amin, Mustafa Farhad, John F. Camilleri‐Brennan, Andrew G N Robertson, Thelma Xerri, Joanna Swann, James Richards, Jasim Amin, Aijaz Jabbar, Phoebe De Bono, Myranda Attard, Hannah Burns, Euan Macdonald, Matthew Baldacchino, Jennifer Skehan, Julian Camilleri‐Brennan, Tom Falconer Hall, Madelaine Gimzewska, Greta Mclachlan, Jamie Shah, James Giles, Selina Chiu, Beatrix Weber, Selina Man Yeng Chiu, Saskia Highcock, Maleeha Hassan, William Beasley, Apostolos Vlachogiorgos, Stephen Dias, Geta Maharaj, Rosie Mcdonald, Alisdair Macdonald, Paul Witherspoon, Alan Baird, Panchali Sarmah, Nikki Green, Haney Youssef, Kate Cross, Clare M Rees, Bernard Van Duren, Emma Upchurch, Khurram Khan, Haytham Abudeeb, Ahmed Hammad, Sharad Karandikar, Doug Bowley, Ahmed Karim, Witold Chachulski, Liam Richardson, Giles Dawnay, Ben Thompson, Ajayesh Mistry, Aneel Bhangu, Millika Ghetia, Sudipta Roy, Ossama Al‐Obaedi, Millika Ghetia, Kaustuv Das, Ash Prabhudesai, DM Cocker, Jessica Juliana Tan, Robert Tyler, Filippo Di Franco, Shruti Ayyar, Sayinthen Vivekanantham, Shyam Gokani, Michael Gillespie, Katrin Gudlaugsdottir, Theodore Pezas, Chelise Currow, Matthew Young‐Han Kim, Amerdip Birring, Joanne Edwards, Ased Ali, Suparna Das, Madan Jha, Kieran Atkinson, Joshua Luck, Thomas Fozard, Michael Puttick, Yahya Salama, Rohi Shah, Ahmad Aboelkassem Ibrahem, Hamdi Ebdewi, Gianpiero Gravante, Saleem El‐Rabaa, Henry Nnajiuba, Rebecca Allott, Aman Bhargava, Zoe Chan, Zaffar Hassan, Misty Makinde, David Hemingway, Ramzana Dean, Alexander Boddy, Ahmed Aber, Vijay Patel, Jehangirshaw Parakh, Sunil Parthiban, Harmony Kaur Ubhi, Simon‐Peter Hosein, Simon Ward, Kamran Malik, Leifa Jennings, Tom Newton, Mirna Alkhouri, Min Kyu Kang, Christopher Houlden, Jonathan Barry, Imtanan Raza, Alistair Farquharson, Sanjeet Bhattacharya, William Milligan, Kate Chang, Liam Henderson, Michael S J Wilson, Yan Ning Neo, Ibrahim Ibrahim, Emily Chan, Fraser S Peck, Pei J Lim, Alexander S North, Rebecca Blundell, Adam Williamson, Dina Fouad, Ashish Minocha, Kathryn Mccarthy, Emma Court, Alice Chambers, Jenna Yee, Ji Chung Tham, Ceri Beaton, Una Walsh, Joseph Lockey, Salman Bokhari, Lara Howells, Megan Griffiths, Laura Yallop, Shailinder Singh, Omar Nasher, Paul Jackson, Michael Puttick, Joshua Luck, Thomas Fozard, Abdul Muiz Shariffuddin, Weng Chee Ho, Michael Sj Wilson, Gurpreet Pabla, Saed Ramzi, Shady Zeidan, Jennifer Doughty, Sidhartha Sinha, Ross Davenport, Jason Lewis, Leo Duffy, Elizabeth Mcaleer, Eleanor Williams, Robin Som, Omar Javed, Matthew Boal, Nicola Harrison, Habib Tafazal, Omar Javed, Tom Brogden, Dmitri Nepogodiev, Ewen Griffiths Rhalumi Daniel Obute, Thomas E Glover, David J Clark, Mohamed Boshnaq, Mansoor Akhtar, Pascale Capleton, Samer Doughan, Mohamed Rabie, Ismail Mohamed, Duncan Samuel, Lauren Dickson, Matthew Kennedy, Eleanor Dempster, Emma Brown, Natalie Maple, Eimear Monaghan, Bernhard Wolf, Alicia Garland, Arthur Mcphee, David Anderson, Robert Anderson, Sarah Hassan, Paul Sutton, Dave Smith, Jonathan Lund, Catherine Boereboom, Jennifer Murphy, Gillian Tierney, Samson Tou, Eleanor Franziska Zimmermann, Neil James Smart, Andrea Marie Warwick, Theodora Stasinou, Ian Daniels, Kim Findlay‐Cooper, Stefan Mitrasinovic, Swayamjyoti Ray, Massimo Varcada, Rovan D'Souza, Sharif Omara, Matthew Spurr, Lucienne Parkinson, Anthony Hanks, Jennifer Ma, Emily Abington, Meera Ramcharn, Gethin Williams, Joseph Winstanley, Ewan D. Kennedy, Emily NW Yeung, Stuart J Fergusson, Catrin Jones, Stephen O'neill, Shujing Jane Lim, Ignatius Liew, Hari Nair, Cameron Fairfield, Julia Oh, Samantha Koh, Andrew Wilson, Catherine Fairfield, Delran Anandkumar, Ashok Kirupagaran, Timothy F Jones, Hew Dt Torrance, Alexander J Fowler, Charmilie Chandrakumar, Priyank Patel, Syed Faaz Ashraf, Sonam M. Lakhani, Aaron Lawson Mclean, Sonia Basson, Jeremy Batt, Catriona Bowman, Michael Stoddart, Natasha Benons, Clare Mason, Rebecca Harrison, John Quayle, Tom Barker, Virginia Summerour, Edward Harper, Caroline Smith, Matthew Hampton, Sophie K Pitt, Alex E Ward, Timothy O'Connor, Emily G Heywood, Thomas M Drake, Abeed Chowdhury, Sina Hossaini, Nicholas Fs Watson, Doug Mckechnie, Ayaan Farah, Anita Chun, Hoey Koh, Grace Lim, Graham Sunderland, Laura Gould, Alice Chambers, P C Munipalle, H Rooney, D R L Browning, Bernadette Pereira, Kristof Nemeth, Emily Decker, Stefano Giuliani, Aly Shalaby, Shafaque Shaikh, Chern Yan Tan, Ebrahim Y A Palkhi, Aleksandra Szczap, Swathikan Chidambaram, Chee Yang Chen, Kavian Kulasabanathan, Srishti Chhabra, Elisabeth Kostov, Philippe Harbord, James Barnacle, Madan Mohan Palliyil, Mina Zikry, Johnathan Porter, Charef Raslan, Mohammed Saeed, Shazia Hafiz, Niksa Soltani, Katie Baillie, Priyanka Singh, Shailee Sheth, Kishen Patel, Mahry Khalili, Jeesoo Choi, Matthew Benger, Lucy Marples, Alastair Macfarlane, Ramesh Thurairaja, Tamsin Boyce, Harriet Whewell, Elin Jones, Francesca Th'ng, Nichola Robertson, Ahmad Mirza, Haroon Saeed, Simon Galloway, Gia Elena, Mohammad Afzal, Mohamed Zakir, Peter Sodde, Charles Hand, Aiesha Sriram, Tamsyn Clark, Patrick Holton, Amy Livesey, Yashashwi Sinha, Fahad Mujtaba Iqbal, Indervir Singh Bharj, Adriana Rotundo, Cara Jenvey, Robert Slade, David Golding, Samuel Haines, Ali Adel Ne'ma Abdullah, Thomas W Tilston, Dafydd Loughran, Danielle Donoghue, Lorenzo Giacci, Mohamed Ashur Sherif, Peter Harrison, Alethea Tang, Deevia Kotecha, Mohamed Elshaer, Tomas Urbonas, Amjid Riaz, Annie Chapman, Parisha Acharya, Joseph Shalhoub, Cathleen Grossart, David McMorran, Makhosini Mlotshwa, William Hawkins, Sofronis Loizides, Kandaswamy Krishna, Melanie Orchard, Chik Wai Ho, Peter Thomson, Shahab Khan, Fiona Taylor, Jalak Shukla, Emma Elizabeth Howie, Linda Macdonald, Olusegun Komolafe, Neil Mcintyre, James Cragg, Jody Parker, Duncan Stewart, Luke Lintin, Julia Tracy, Tahir Farooq, George Molina, Haytham Kaafarani, Laura Luque, Robel Beyene, Jack Sava, Mark Scott, Mamta Swaroop, Raelene Kennedy, Ijeoma A Azodo, Daithi Heffernan, Tristen Chun, Andrew Stephen, Melanie Sion, Michael S. Weinstein, Viren Punja, Nikolay Bugaev, Monica Goodstein, Shadi Razmdjou, Eric Etchill, Juan Carlos Puyana, Matthew Kesinger, Lena Napolitano, Kathleen To, Mark Hemmila, Oliver Todd, Edward Jenner, Ellen Hoogakker, Besmir Grizhja, Shpetim Ymeri, Gezim Galiqi, Roberto Klappenbach, Diego Antezana, Alvaro Enrique Mendoza Beleño, Cecilia Costa, Belen Sanchez, Susan Aviles, Maria Marta Modolo, Claudio Gabriel Fermani, Rubén Balmaceda, Santiago Villalobos, Juan Manuel Carmona, Daniel Hamill, Peter Deutschmann, Simone Sandler, Daniel Cox, Ram Nataraja, Claire Sharpin, Damir Ljuhar, Demi Gray, Morgan Haines, Dush Iyer, Nithya Niranjan, Scott D'Amours, Morvarid Ashtari, Helena Franco, Ashrarur Rahman Mitul, Sabbir Karim, Nowrin F. Aman, Mahnuma Mahfuz Estee, Umme Salma, Joyeta Razzaque, Tasnia Hamid Kanta, Sayeeda Aktar Tori, Shadid Alamin, Swapnil Roy, Shadid Al Amin, Rezaul Karim, Muhtarima Haque, Amreen Faruq, Farhana Iftekhar, Margaret O'Shea, Greg Padmore, Ramesh Jonnalagadda, Andrey Litvin, Aliaksandr Filatau, Dzmitry Paulouski, Maryna Shubianok, Tatsiana Shachykava, Dzianis Khokha, Vladimir Khokha, Fernande Djivoh, Lawani Ismaïl, Francis Dossou, Djifid Morel Seto, Dansou Gaspard Gbessi, Bruno Noukpozounkou, Yacoubou Imorou Souaibou, Kpèmahouton René Keke, Fred Hodonou, Ernest Yemalin Stephane Ahounou, Thierry Alihonou, Max Dénakpo, Germain Ahlonsou, Alemayehu Ginbo Bedada, Carlos Nsengiyumva, Sandrine Kwizera, Venerand Barendegere, Philip Choi, Simon Stock, Luai Jamal, Mohammed Firdouse, Augusto Zani, Georges Azzie, Sameer Kushwaha, Arnav Agarwal, Tzu‐Ling Chen, Chingwan Yip, Irene Montes, Felipe Zapata, Sebastian Sierra, Maria Isabel Villegas Lanau, Maria Clara Mendoza Arango, Ivan Mendoza Restrepo, Sebastian Sierra, Ruben Santiago Restrepo Giraldo, Maria Clara Mendoza Arango, Edgar Domini, Robert Karlo, Jakov Mihanovic, Mohamed Youssef, Hossam Elfeki, Waleed Thabet, Aly Sanad, Gehad Tawfik, Ahmed Zaki, Noran Abdel‐Hameed, Mohamed Mostafa, Muhammad Fathi Waleed Omar, Ahmed Ghanem, Emad Abdallah, Adel Denewar, Eman Emara, Eman Rashad, Ahmad Sakr, Rehab Elashry, Sameh Emile, Toqa Khafagy, Sara Elhamouly, Arwa Elfarargy, Amna Mamdouh Mohamed, Ghada Saied Nagy, Abeer Esam, Eman Elwy, Aya Hammad, Salwa Khallaf, Eman Ibrahim, Ahmed Saidbadr, Ahmed Moustafa, Amany Eldosouky Mohammed, Mohammed Elgheriany, Eman Abdelmageed, Eman Abd Al Raouf, Esraa Samir Elbanby, Maha Elmasry, Mahitab Morsy Farahat, Eman Yahya Mansor, Eman Magdy Hegazy, Esraa Gamal, Heba Gamal, Hend Kandil, Doaa Maher Abdelrouf, Mohamed Moaty, Dina Gamal, Nada El‐Sagheer, Mohamed Salah, Salma Magdy, Asmaa Salah, Ahmed Essam, Ahmed Ali, Mahmoud Badawy, Sara Ahmed, Mazed Mohamed, Abdelrahman Assal, Mohamed Sleem, Mai Ebidy, Aly Abd Elrazek, Diaaaldin Zahran, Nourhan Adam, Mohamed Nazir, Adel B Hassanein, Ahmed Ismail, Amira Elsawy, Rana Mamdouh, Mohamed Mabrouk, Lopna Ahmed Mohamed Ahmed, Mohamed Hassab Alnaby, Eman Magdy, Manar Abd‐Elmawla, Marwan Fahim, Bassant Mowafy, Moustafa Ibrahim Mahmoud, Meran Allam, Muhammad Alkelani, Noran Halim El Gendy, Mariam Saad Aboul‐Naga, Reham Alaa El‐Din, Alyaa Halim Elgendy, Mohamed Ismail, Mahmoud Shalaby, Aya Adel Elsharkawy, Mahmoud Elsayed Moghazy, Khaled Hesham Elbisomy, Hend Adel Gawad Shakshouk, Mohamed Fouad Hamed, Mai Mohamed Ebidy, Mostafa Abdelkader, Mohamed Karkeet, Hayam Ahmed, Israa Adel, Mohammad Elsayed Omar, Mohamed Ibrahim, Omar Ghoneim, Omar Hesham, Shimaa Gamal, Karim Hilal, Omar Arafa, Sawsan Adel Awad, Menatalla Salem, Fawzia Abdellatif Elsherif, Nourhan Elsabbagh, Moustafa R. Aboelsoud, Ahmed Hossam Eldin Fouad Rida, Amr Hossameldin, Ethar Hany, Yomna Hosny Asar, Nourhan Anwar, Mohamed Gadelkarim, Samar Abdelhady, Eman Mohamed Morshedy, Reham Saad, Nourhan Soliman, Mahmoud Salama, Eslam Ezzat, Arwa Mohamed, Arwa Ibrahim, Alaa Fergany, Sara Mohammed, Aya Reda, Yomna Allam, Hanan Adel Saad, Afnan Abdelfatah, Aya Mohamed Fathy, Ahmed El‐Sehily, Esraa Abdalmageed Kasem, Ahmed Tarek Abdelbaset Hassan, Ahmed Rabeih Mohammed, Abdalla Gamal Saad, Yasmin Elfouly, Nesma Elfouly, Arij Ibrahim, Amr Hassaan, Mohammed Mustafa Mohammed, Ghada Elhoseny, Mohamed Magdy, Esraa Abd Elkhalek, Yehia Zakaria, Tarek Ezzat, Ali Abo El Dahab, Mohamed Kelany, Sara Arafa, Osama Mokhtar Mohamed Hassan, Nermin Mohamed Badwi, Ahmad Saber Sleem, Hussien Ahmed, Kholoud Abdelbadeai, Mohamed Abozed Abdullah, Muhammad Amsyar Auni Lokman, Suraya Bahar, Anan Rady Abdelazeam, Abdelrahman Adelshone, Muhammad Bin Hasnan, Athirah Zulkifli, Siti Nur Alia Kamarulzamil, Abdelaziz Elhendawy, Aliang Latif, Ahmad Bin Adnan, Shahadatul Shaharuddin, Aminah Hanum Haji Abdul Majid, Mahmoud Amreia, Dina Al‐Marakby, Mahmoud Salma, Mohamad Jeffrey Bin Ismail, Elissa Rifhan Mohd Basir, Citra Dewi Mohd Ali, Aya Yehia Ata, Maha Nasr, Asmaa Rezq, Ahmed Sheta, Sherif Tariq, Abd Elkhalek Sallam, Abdelrhman KZ Darwish, Sohaila Elmihy, Shady Elhadry, Ahmed Farag, Haidar Hajeh, Abdelaziz Abdelaal, Amro Aglan, Ahmed Zohair, Mahitab Essam, Omar Moussa, Esraa El‐Gizawy, Mostafa Samy, Safia Ali, Esraa Elhalawany, Ahmed Ata, Mohamed El Halawany, Mohamed Nashat, Samar Soliman, Alaa Elazab, Mostada Samy, Mohamed A Abdelaziz, Khaled Ibrahim, Ahmed mohamed Ibrahim, Ammar Gado, Usama Hantour, Esraa Alm Eldeen, Mohamed Reda loaloa, Arwa Abouzaid, Mostafa Ahmed Bahaa Eldin, Eman Hashad, Fathy Sroor, Doaa Gamil, Eman Mahmoud Abdulhakeem, Mahmoud Zakaria, Fawzy Mohamed, Marwan Abubakr, Elsayed Ali, Hesham Magdy, Ahmed Refaat, Menna Tallah Ramadan, Mohamed Abdelaty Mohamed, Salma Mansour, Hager Abdul Aziz Amin, Ahmed Rabie Mohamed, Mahmoud Saami, Nada Ahmed Reda Elsayed, Adham Tarek, Sabry Mohy Eldeen Mahmoud, Islam Magdy El Sayed, Amira Reda, Martina Yusuf Shawky, Mohammed Mousa Salem, Shahinaz Alaa El‐Din, Noha Abdullah Soliman, Muhammed Talaat, Shahinaz Alaael‐Dein, Ahmed Abd Elmoen Elhusseiny, Noha Abdullah, Mohammed Elshaar, Aya Abdel Fatah Ibraheem, Hager Abdulaziz, Mohammed Kamal Ismail, Mona Hamdy Madkor, Mohamed Abdelaty, Sara Mahmoud Abdel‐Kader, Osama Mohamed Salah, Mahmoud Eldafrawy, Ahmed Zaki Eldeeb, Mostafa Mahmoud Eid, Attia Attia, Khalid Salah El‐Dien, Ayman Shwky, Mohamed Adel Badenjki, Abdelrahman Soliman, Samaa Mahmoud Al Attar, Farrag Sayed, Fahd Abdel Sabour, Mohammed G. Azizeldine, Muhammad Shawqi, Abdullah Hashim, Ahmed Aamer, Ahmed Mahmoud Abdelraouf, Mahmoud Abdelshakour, Amal Ibrahim, Basma Mahmoud, Mohamed Ali Mahmoud, Mostafa Qenawy, Ahmed M. Rashed, Ahmed Dahy, Marwa Sayed, Ahmed W. Shamsedine, Bakeer Mohamed, Ahmad Hasan, Mahmoud M. Saad, Khalil Abdul Bassit, Nadia Khalid Abd El‐Latif, Nada Elzahed, Ahmed El Kashash, Nada Mohamed Bekhet, Sarah Hafez, Ahmed Gad, Mahmoud Elkhadragy Maher, Ahmed Abd Elsameea, Mohamed Hafez, Ahmad Sabe, Ataa Ahmed, Ahmed Shahine, Khaled Dawood, Shireen Gaafar, Reem Husseiny, Omnia Aboelmagd, Ahmed Soliman, Nourhan Mesbah, Hossam Emadeldin, Amgad Al Meligy, Amira Hassan Bekhet, Doaa Hasan, Khaled Alhady, Ahmad Khaled Sabe, Mahmoud A. Elnajjar, Majed Aboelella, Ward Hamsho, Ihab Hassan, Hala Saad, Galaleldin Abdelazim, Hend Mahmoud, Noha Wael, Ahmedali M Kandil, Ahmed Magdy, Shimaa Said Elkholy, Badr Eldin Adel, Kareem Dabbour, Saged Elsherbiney, Omar Mattar, Abdulshafi Khaled Abdrabou, Mohammed Yahia Mohamed Aly, Abdelrahman Geuoshy, Ahmedglal Elnagar, Saraibrahim Ahmed, Ibrahem Abdelmotaleb, Amr Ahmed Saleh, Manar Saeed, Shady Mahmoud, Badreldin Adel Tawfik, Samar Adel Ismail, Esraay Zakaria, Mariam O. Gad, Mohamed Salah Elhelbawy, Monica Bassem, Noha Maraie, Nourhan Medhat Elhadary, Nourhan Semeda, Shaza Rabie Mohamed, Hesham Mohammed Bakry, AA Essam, Dina Tarek, Khlood Ashour, Alaa Elhadad, Abdulrahman Abdel‐Aty, Ibrahim Rakha, Sara Mamdouh Matter, Rasha Abdelhamed, Omar Abdelkader, Ayat Hassaan, Yasmin Soliman, Amna Mohamed, Sara Ghanem, Sara Amr Mohamed Farouk, Eman Mohamed Ibrahim, Esraa El‐Taher, Merna Mostafa, Mohamed Fawzy Mahrous Badr, Rofida Elsemelawy, Aya El‐Sawy, Ahmad Bakr, Ahmad Abdel Razaq Al Rafati, Sten Saar, Arvo Reinsoo, Peep Talving, Nebyou Seyoum, Tewodros Worku, Agazi Fitsum, Matti Tolonen, Ari Leppäniemi, Ville Sallinen, Benoît Parmentier, Matthieu Peycelon, Sabine Irtan, Sabrina Dardenne, Elsa Robert, Betty Maillot, Etienne Courboin, Alexis Pierre Arnaud, Juliette Hascoet, Olivier Abbo, Amir Ait Kaci, Thomas Prudhomme, Quentin Ballouhey, Céline Grosos, Laurent Fourcade, Tolg Cecilia, Colombani Jean‐Francois, Francois‐Coridon Helene, Xavier Delforge, Elodie Haraux, Bertrand Dousset, Roberto Schiavone, Sebastien Gaujoux, Jean‐Baptiste Marret, Aurore Haffreingue, Julien Rod, Mariette Renaux‐Petel, Jean‐François Lecompte, Jean Bréaud, Pauline Gastaldi, Chouikh Taieb, Raquillet Claire, Echaieb Anis, Nasir Bustangi, Manuel Lopez, Aurelien Scalabre, Maria Giovanna Grella, Aurora Mariani, Guillaume Podevin, Françoise Schmitt, Erik Hervieux, Aline Broch, Cecile Muller, Stephen Tabiri, Anyomih Theophilus Teddy Kojo, Dickson Bandoh, Francis Abantanga, Martin Kyereh, Hamza Asumah, Eric Kofi Appiah, Paul Wondoh, Adam Gyedu, Charles Dally, Kwabena Agbedinu, Michael Amoah, Abiboye Yifieyeh, Frank Owusu, Mabel Amoako‐Boateng, Makafui Dayie, Richmond Hagan, Sam Debrah, Micheal Ohene‐Yeboah, Joe‐Nat Clegg‐Lampety, Victor Etwire, Jonathan Dakubo, Samuel Essoun, William Bonney, Hope Glover‐Addy, Samuel Osei‐Nketiah, Joachim Amoako, Niiarmah Adu‐Aryee, William Appeadu‐Mensah, Antoinette Bediako‐Bowan, Florence Dedey, Mattew Ekow, Emmanuel Akatibo, Musah Yakubu, Hope Edem Kofi Kordorwu, Kwasi Asare‐Bediako, Enoch Tackie, Kenneth Aaniana, Emmanuel Acquah, Richard Opoku‐Agyeman, Anthony Avoka, Kwasi Kusi, Kwame Maison, Frank Enoch Gyamfi, Gandau Naa Barnabas, Saiba Abdul‐Latif, Philip Taah Amoako, Anthony Davor, Victor Dassah, Enoch Dagoe, Prince Kwakyeafriyie, Elliot Akoto, Eric Ackom, Ekow Mensah, Ebenezer Takyi Atkins, Christian Lari Coompson, Nikolaos Ivros, Christoforos Ferousis, Vasileios Kalles, Christos Agalianos, Ioannis Kyriazanos, Christos Barkolias, Angelos Tselos, Georgios Tzikos, Evangelos Voulgaris, Dimitrios Lytras, Athanasia Bamicha, Kyriakos Psarianos, Anastasios Stefanopoulos, Ioannis Patoulias, Dimitrios Sfougaris, Ioannis Valioulis, Dimitrios Balalis, Dimitrios Korkolis, Dimitrios K Manatakis, Georgios Kyrou, Georgios Karabelias, Iason‐Antonios Papaskarlatos, Kolonia Konstantina, Nikolaos Zampitis, Stylianos Germanos, Aspasia Papailia, Theodosios Theodosopoulos, Georgios Gkiokas, Magdalini Mitroudi, Christina Panteli, Thomas Feidantsis, Konstantinos Farmakis, Dimitrios Kyziridis, Orestis Ioannidis, Styliani Parpoudi, Georgios Gemenetzis, Stavros Parasyris, Christos Anthoulakis, Nikolaos Nikoloudis, Michail Margaritis, Maria‐Lorena Aguilera‐Arevalo, Otto Coyoy‐Gaitan, Javier Rosales, Luis Tale, Rafael Soley, Emmanuel Barrios, Servio Tulio Torres Rodriguez, Carlos Paz Galvez, Danilo Herrera Cruz, Guillermo Sanchez Rosenberg, Alejandro Matheu, David Monterroso Cohen, Marie Paul, Angeline Charles, Justin Chak Yiu Lam, Man Hon Andrew Yeung, Chi Ying Jacquelyn Fok, Ka Hin Gabriel Li, Anthony Chuk‐Him Lai, Yuk Hong Eric Cheung, Hong Yee Wong, Ka Wai Leung, Tien Seng Bryan Lee, Wai Him Lam, Weihei Dao, Stephanie Hiu‐wai Kwok, Tsz‐Yan Katie Chan, Yung Kok Ng, TWC Mak, Qinyang Liu, Chi Chung Foo, James Yang, Basant Kumar, Ankur Bhatnagar, Vijaid Upadhyaya, Sunil Kumar, Uday Muddebihal, Wasim Dar, KC Janardha, Philip Alexander, Neerav Aruldas, Fidelis Jacklyn Adella, Anthonius Santoso Rulie, Ferdy Iskandar, Jonny Setiawan, Cicilia Viany Evajelista, Hani Natalie, Arlindawati Suyadi, Rudy Gunawan, Herlin Karismaningtyas, Lusi Padma Sulistianingsih Mata, Ferry Fitriya Ayu Andika, Afifatun Hasanah, T Ariani Widiastini, Nurlaila Ayu Purwaningsih, Annisa Dewi Fitriana Mukin, Dina Faizatur Rahmah, Hazmi Dwinanda Nurqistan, Hasbi Maulana Arsyad, Novia Adhitama, Wifanto Saditya Jeo, Nathania Sutandi, Audrey Clarissa, Phebe Anggita Gultom, Matthew Billy, Andreass Haloho, Radhian Amandito, Nadya Johanna, Felix Lee, Radin Mohd Nurrahman Radin Dorani, Martha Glynn, Mohammad Alherz, Wennweoi Goh, Haaris A. Shiwani, Lorraine Sproule, Kevin C. Conlon, Miklosh Bala, Asaf Kedar, Luca Turati, Federica Bianco, Francesca Steccanella, Gaetano Gallo, Mario Trompetto, Giuseppe Clerico, Matteo Papandrea, Giuseppe Sammarco, Rosario Sacco, Angelo Benevento, Francesco Pata, Luisa Giavarini, Mariano Cesare Giglio, Luigi Bucci, Gianluca Pagano, Viviana Sollazzo, Roberto Peltrini, Gaetano Luglio, Arianna Birindelli, Salomone Di Saverio, Gregorio Tugnoli, Miguel Angel Paludi, Pietro Mingrone, Domenica Pata, Francesco Selvaggi, Lucio Selvaggi, Gianluca Pellino, Natale Di Martino, Gianluca Curletti, Paolo Aonzo, Raffaele Galleano, Stefano Berti, Elisa Francone, Silvia Boni, Laura Lorenzon, Annalisa lo Conte, Genoveffa Balducci, Gianmaria Confalonieri, Giovanni Pesenti, Laura Gavagna, Giorgio Vasquez, Simone Targa, Savino Occhionorelli, Dario Andreotti, Giacomo Pata, Andrea Armellini, Deborah Chiesa, Fabrizio Aquilino, Nicola Chetta, Arcangelo Picciariello, Mohamed Abdelkhalek, Andrea Belli, Silvia De Franciscis, Annamaria Bigaran, Alessandro Favero, Stefano M.M Basso, Paola Salusso, Martina Perino, Sylvie Mochet, Diego Sasia, Francesco Riente, Marco Migliore, David Merlini, Silvia Basilicò, Carlo Corbellini, Veronica Lazzari, Yuri Macchitella, Luigi Bonavina, Daniele Angelieri, Diego Coletta, Federica Falaschi, Marco Catani, Claudia Reali, Mariastella Malavenda, Celeste Del Basso, Sergio Ribaldi, Massimo Coletti, Andrea Natili, Norma Depalma, Immacolata Iannone, Angelo Antoniozzi, Davide Rossi, Daniele Gui, Gerardo Perrotta, Matteo Ripa, Francesco Ruben Giardino, Maurizio Foco, Erika Vicario, Federico Coccolini, Luca Ansaloni, Gabriela Elisa Nita, Nicoletta Leone, Andrea Bondurri, Anna Maffioli, Andrea Simioni, Davide De Boni, Sandro Pasquali, Elena Goldin, Elena Vendramin, Eleonora Ciccioli, Umberto Tedeschi, Luca Bortolasi, Paola Violi, Tommaso Campagnaro, Simone Conci, Giovanni Lazzari, Calogero Iacono, Alfredo Gulielmi, Serena Manfreda, Anna Rinaldi, Maria Novella Ringressi, Beatrice Brunoni, Giuseppe Salamone, Mirko Mangiapane, Paolino De Marco, Antonella La Brocca, Roberta Tutino, Vania Silvestri, Leo Licari, Tommaso Fontana, Nicolò Falco, Gianfranco Cocorullo, Mostafa Shalaby, Pierpaolo Sileri, Claudio Arcudi, Isam Bsisu, Khaled Aljboor, Lana Abusalem, Aseel Alnusairat, Ahmad Qaissieh, Emad Al‐Dakka, Ali Ababneh, Oday Halhouli, Taha Yusufali, Hussein Mohammed, Justus Lando, Robert Parker, Wairimu Ndegwa, Mantas Jokubauskas, Jolanta Gribauskaite, Donatas Venskutonis, Justas Kuliavas, Audrius Dulskas, Narimantas E. Samalavicius, Kristijonas Jasaitis, Audrius Parseliunas, Viktorija Nevieraite, Margarita Montrimaite, Evelina Slapelyte, Edvinas Dainius, Romualdas Riauka, Zilvinas Dambrauskas, Andrejus Subocius, Linas Venclauskas, Antanas Gulbinas, Saulius Bradulskis, Simona Kasputyte, Deimante Mikuckyte, Mindaugas Kiudelis, Justas Zilinskas, Tomas Jankus, Steponas Petrikenas, Matas Pažuskis, Zigmantas Urniežius, Mantas Vilčinskas, Vincas Jonas Banaitis, Vytautas Gaižauskas, Edvard Grisin, Povilas Mazrimas, Rokas Rackauskas, Mantas Drungilas, Karolis Lagunavicius, Vytautas Lipnickas, Dovilè Majauskyté, Valdemaras Jotautas, Tomas Abaliksta, Laimonas Uščinas, Gintaras Simutis, Adomas Ladukas, Donatas Danys, Erikas Laugzemys, Saulius Mikalauskas, Tomas Poškus, Elena Zdanyte Sruogiene, Petras Višinskas, Reda Žilinskienė, Deividas Dragatas, Andrius Burmistrovas, Zygimantas Tverskis, Arturas Vaicius, Ruta Mazelyte, Antanas Zadoroznas, Nerijus Kaselis, Greta Žiubrytė, Finaritra Casimir Fleur Prudence Rahantasoa, Luc Hervé Samison, Fanjandrainy Rasoaherinomenjanahary, Todisoa Emmanuella Christina Tolotra, Cornelius Mukuzunga, Vanessa Msosa, Chimwemwe Kwatiwani, Nelson Msiska, Feng Yih Chai, Siti Mohd Desa Asilah, Khuzaimah Zahid Syibrah, Pui Xin Chin, Afizah Salleh, Nur Zulaika Riswan, April Camilla Roslani, Hoong‐Yin Chong, Nora Abdul Aziz, Keat‐Seong Poh, Chu‐Ann Chai, Sandip Kumar, Mustafa Mohammed Taher, Nik Ritza Kosai, Dayang Nita Abdul Aziz, Reynu Rajan, Rokayah Julaihi, Durvesh Lacthman Jethwani, Muhammad Taqiyuddin Yahaya, Nik Azim Nik Abdullah, Susan Wndy Mathew, Kuet Jun Chung, Milaksh Kumar Nirumal, R. Goh Ern Tze, Syed Abdul Wahhab Eusoffee Wan Ali, Yiing Yee Gan, Jesse Ron Swire Ting, Samuel S. Y. Sii, Kean Leong Koay, Yi Koon Tan, Alvin Ee Zhiun Cheah, Chui Yee Wong, Tuan Nur'Azmah Tuan Mat, Crystal Yern Nee Chow, Prisca A.L. Har, Yishan Der, Yong Yong Tew, Fitjerald Henry, Xinwei Low, Ya Theng Neo, Hian Ee Heng, Shu Ning Kong, Cheewei Gan, Yi Ting Mok, Yee Wen Tan, Kandasami Palayan, Mahadevan Deva Tata, Yih Jeng Cheong, Kuhaendran Gunaseelan, Wan Nurul Ain Wan Mohd Nasir, Pigeneswaren Yoganathan, Eu Xian Lee, Jian Er Saw, Li Jing Yeang, Pei Ying Koh, Shyang Yee Lim, Shuang Yi Teo, Nicole Grech, Daniela Magri, Kristina Cassar, Christine Mizzi, Malcolm Falzon, Nihaal Shaikh, Ruth Scicluna, Stefan Zammit, Elaine Borg, Sean Mizzi, Svetlana Doris Brincat, Thelma Tembo, Vu Thanh Hien Le, Tara Grima, Keith Sammut, Kurt Carabott, Alexia Farrugia, Ciskje Zarb, Andre Navarro, Thea Dimech, Georgette Marie Camilleri, Isaac Bertuello, Jeffrey Dalli, Karl Bonavia, Samantha Corro‐Diaz, Marisol Manriquez‐Reyes, Antonio Ramos‐De la Medina, Amina Abdelhamid, Abdelmalek Hrora, Sarah Benammi, Houda Bachri, Meryem Abbouch, Khaoula Boukhal, Redouane Mammar Bennai, Abdelkader Belkouchi, Mohamed Sobhi Jabal, Chaymae Benyaiche, Maarten Vermaas, Lucia Duinhouwer, Javier Pastora, Greta Wood, Maria Soledad Merlo, Akinlabi Ajao, Omobolaji Ayandipo, Taiwo Lawal, Abdussemiu Abdurrazzaaq, Muslimat Alada, Abdulrasheed Nasir, James Adeniran, Olufemi Habeeb, Ademola Popoola, Ademola Adeyeye, Ademola Adebanjo, Opeoluwa Adesanya, Adewale Adeniyi, Henry Mendel, Bashir Bello, Umar Muktar, Adedapo Osinowo, Thomas Olagboyega Olajide, Oyindamola Oshati, George Ihediwa, Babajide Adenekan, Victor Nwinee, Felix Alakaloko, Adesoji Ademuyiwa, Olumide Elebute, Abdulrazzaq Lawal, Chris Bode, Mojolaoluwa Olugbemi, Alaba Adesina, Olubukola Faturoti, Oluwatomi Odutola, Oluwaseyi Adebola, Clement Onuoha, Ogechukwu Taiwo, Omolara Williams, Fatai Balogun, Olalekan Ajai, Mobolaji Oludara, Iloba Njokanma, Roland Osuoji, Stephen Kache, Jonathan Ajah, Jerry Makama, Ahmed Adamu, Suleiman Baba, Mohammad Aliyu, Shamsudeen Aliyu, Yahaya Ukwenya, Halima Aliyu, Tunde Sholadoye, Muhammad Daniyan, Oluseyi Ogunsua, Lofty‐John Anyanwu, Abdurrahaman Sheshe, Aminu Mohammad, Samson Olori, Philip Mshelbwala, Babatunde Odeyemi, Garba Samson, Oyediran Kehinde Timothy, Sani Ali Samuel, Anthony Ajiboye, Ademola Adeyeye, Isaac Amole, Olajide Abiola, Akin Olaolorun, Kjetil Søreide, Torhild Veen, Arezo Kanani, Kristian Styles, Ragnar Herikstad, Johannes Wiik Larsen, Jon Arne Søreide, Elisabeth Jensen, Mads Gran, Eirik Kjus Aahlin, Tina Gaarder, Peter Wiel Monrad‐Hansen, Pål Aksel Næss, Giedrius Lauzikas, Joachim Wiborg, Silje Holte, Knut Magne Augestad, Gurpreet Singh Banipal, Michela Monteleone, Thomas Tetens Moe, Johannes Kurt Schultz, Taher Al‐taher, Ayah Hamdan, Ayman Salman, Rana Saadeh, Aseel Musleh, Dana Jaradat, Soha Abushamleh, Sakhaa Hanoun, Amjad Abu Qumbos, Aseel Hamarshi, Ayman And Taher, Israa Qawasmi, Khalid Qurie, Marwa Altarayra, Mohammad Ghannam, Alaa Shaheen, Azher Herebat, Aram Abdelhaq, Ahmad Shalabi, Maram Abu‐toyour, Fatema Asi, Ala Shamasneh, Anwar Atiyeh, Mousa Mustafa, Rula Zaa'treh, Majd Dabboor, Enas Alaloul, Heba Baraka, Jehad Meqbil, Alaa Al‐Buhaisi, Mohamedraed Elshami, Samah Afana, Sahar Jaber, Said Alyacoubi, Yousef Abuowda, Tasneem Idress, Eman Abuqwaider, Sara Al‐saqqa, Alaa Bowabsak, Alaa El Jamassi, Doaa Hasanain, Hadeel Al‐farram, Maram Salah, Aya Firwana, Marwa Hamdan, Israa Awad, Ahmad Ashour, Fayez Elian Al Barrawi, Ahmed Al‐khatib, Maha Al‐faqawi, Mohamed Fares, Amjad Elmashala, Mohammad Adawi, Ihdaa Adawi, Reem Khreishi, Rose Khreishi, Ahmad ashour, Ahed Ghaben, Najwa Nadeem, Muhammad Saqlain, Jibran Abbasy, Abdul Rehman Alvi, Tanzeela Gala, Noman Shahzad, Kamran Faisal Bhopal, Zainab Iftikhar, Muhammad Talha Butt, Syed Asaat ul Razi, Asdaq Ahmed, Ali Khan Niazi, Ibrahim Raza, Fatima Baluch, Ahmed Raza, Ahmad Bani‐Sadar, Ahmad Uzair Qureshi, Muhammad Adil, Awais Raza, Mahnoor Javaid, Muhammad Waqar, Maryam Ali Khan, Mohammad Mohsin Arshad, Mohammadasim Amjad, Gustavo Miguel Machain Vega, Jorge Torres Cardozo, Marcelo O'Higgins Roche, Gustavo Rodolfo Pertersen Servin, Helmut Alfredo Segovia Lohse, Larissa Ines Páez Lopez, Ramón Augusto Melo Cardozo, Fernando Espinoza, Angel David Pérez Rojas, Diana Sanchez, Camila Sanchez Samaniego, Shalon Guevara Torres, Alexander Canta Calua, Cesar Razuri, Nadia Ortiz, Xianelle Rodriguez, Nahilia Carrasco, Fridiz Saravia, Hector Shibao Miyasato, María Valcarcel‐Saldaña, Ysabel Esthefany Alejos Bermúdez, Juan Carpio, Walter Ruiz Panez, Pedro Angel Toribio Orbegozo, Carolina Guzmán Dueñas, Kevin Turpo Espinoza, Ana Maria Sandoval Barrantes, Jorge Armando Chungui Bravo, Sebastian Shu, Lorena Fuentes‐Rivera, Carmen Fernández, Diego Romani, Bárbara Málaga, Joselyn Ye, Ricardo Velasquez, Jannin Salcedo, Ana Lucia Contreras‐Vergara, Angelica Genoveva Vergara Mejia, Maria Soledad Gonzales Montejo, Marilia Del Carmen Escalante Salas, Willy Alcca Ticona, Marvin Vargas, George Christian Manrique Sila, Robinson Mas, Arazzelly del Pilar Paucar, Armando José Román Velásquez, Alina Robledo‐Rabanal, Ludwing Alexander Zeta Solis, Kenny Turpo Espinoza, José Luis Hamasaki Hamaguchi, Erick Samuel Florez Farfan, Linda Alvi Madrid Barrientos, Juan Jaime Herrera Matta, John Jemuel V. Mora, Menold Archee P. Redota, Manuel Francisco Roxas, Maria Jesusa B. Maño, Marie Dione Parreno‐Sacdalan, Marie Carmela Lapitan, Christel Leanne Almanon, Maciej Walędziak, Rafał Roszkowski, Michał Janik, Anna Lasek, Piotr Major, Dorota Radkowiak, Mateusz Rubinkiewicz, Cristina Fernandes, Jose Costa‐Maia, Renato Melo, Liviu Muntean, Aurel Sandu Mironescu, Lucian Corneliu Vida, Amar Kourdouli, Mariuca Popa, Hogea Mircea, Mihaela Vartic, Bogdan Diaconescu, Matei Razvan Bratu, Ionut Negoi, Mircea Beuran, Cezar Ciubotaru, J.C Allen Ingabire, Alphonse Zeta Mutabazi, Norbert Uzabumwana, Dieudonne Duhoranenayo, Elio Jovine, Nicola Zanini, Giovanni Landolfo, Murad Aljiffry, Faisal Idris, Mohammed Saleh A. Alghamdi, Ashraf Maghrabi, Abdulmalik Altaf, Aroub Alkaaki, Ahmad Khoja, Abrar Nawawi, Sondos Turkustani, Eyad Khalifah, Ahmad Gudal, Adel Albiety, Sarah Sahel, Reham Alshareef, Mohammed Najjar, Ahmed Alzahrani, Ahmed Alghamdi, Wedyan Alhazmi, Ghiath Al Saied, Mohammed Alamoudi, Muhammed Masood Riaz, Mazen Hassanain, Basmah Alhassan, Abdullah Altamimi, Reem Alyahya, Norah Al Subaie, Fatema Al Bastawis, Afnan Altamimi, Thamer Nouh, Roaa Khan, Milan Radojkovic, Ljiljana Jeremic, Milica Nestorovic, Jia Hao Law, Keith Say Kwang Tan, Ryan Choon Kiat Tan, Joel Kin Tan, Lau Wen Liang Joel, Bettina Lieske, Xue Wei Chan, Faith Qi Hui Leong, Choon Seng Chong, Sharon Koh, Kai Yin Lee, Kuok Chung Lee, Kent Pluke, Britta Dedekind, Puyearashid Nashidengo, Mark Ian Hampton, Johanna Joosten, Sanju Sobnach, Liana Roodt, Anthony Sander, James Pape, Richard Spence, Niveshni Maistry, Phumudzo Ndwambi, Kamau Kinandu, Myint Tun, Frederick Du Toit, Quinn Ellison, Sule Burger, DC Grobler, Lawrence Bongani Khulu, Rachel Moore, Vicky Jennings, Astrid Leusink, Nazmie Kariem, Juan Gouws, Kathryn Chu, Heather Bougard, Fazlin Noor, Angela Dell, Sarah Rayne, Stephanie Van Straten, Arvin Khamajeet, Serge Kapenda Tshisola, Kalangu Kabongo, Victor Kong, Yoshan Moodley, Frank Anderson, Thandinkosi Madiba, Flip du Plooy, Leila Hartford, Gareth Chilton, Parveen Karjiker, Matlou Ernest Mabitsela, Sibongile Ruth Ndlovu, Maria Badicel, Robert Jaich, Jaime Ruiz‐Tovar, Luis Garcia‐Florez, Jorge L. Otero‐Díez, Virginia Ramos Pérez, Nuria Aguado Suárez, Javier Minguez García, Sara Corral Moreno, Maria Vicenta Collado, Virginia Jiménez Carneros, Javier García Septiem, Mariana Gonzalez, Antonio Picardo, Enrique Esteban, Esther Ferrero, Irene Ortega, Eloy Espin‐Basany, Ruth Blanco‐Colino, Valeria Andriola, Lorena Solar García, Elisa Contreras, Carmen García Bernardo, Janet Pagnozzi, Sandra Sanz, Alberto Miyar de León, Asnel Dorismé, Joseluis Rodicio, Aida Suarez, Jessica Stuva, Tamara Diaz Vico, Laura Fernandez‐Vega, Carla Soldevila‐Verdeguer, Fatima Sena‐Ruiz, Natalia Pujol‐Cano, Paula Diaz‐Jover, José Maria Garcia‐Perez, Juan Jose Segura‐Sampedro, Cristina Pineño‐Flores, David Ambrona‐Zafra, Andrea Craus‐Miguel, Patricia Jimenez‐Morillas, Angela Mazzella, A.B Jayathilake, S.P.B Thalgaspitiya, L.S. Wijayarathna, P.M.S.N. Wimalge, Hakeem Ayomi Sanni, Aliyu Ndajiwo, Ogheneochuko Okenabirhie, Anmar Homeida, Abobaker Younis, Omer Abdelbagi Omer, Mustafa Abdulaziz, Ali Mussad, Ali Adam, Yucel Cengiz, Ida Björklund, Sandra Ahlqvist, Sandra Ahlqvist, Anders Thorell, Fredrik Wogensen, Arestis Sokratous, Michaela Breistrand, Hildur Thorarinsdottir, Johanna Sigurdadottir, Maziar Nikberg, Abbas Chabok, Maria Hjertberg, Peter Elbe, Deborah Saraste, Wiktor Rutkowski, Louise Forlin, Karoliina Niska, Malin Sund, Dennis Oswald, Georgios Peros, Rafael Bluelle, Katharina Reinisch, Daniel Frey, Adrian Palma, Dimitri Aristotle Raptis, Lucius Zumbühl, Markus Zuber, Roger Schmid, Gabriela Werder, Antonio Nocito, Alexandra Gerosa, Silke Mahanty, Lukas Werner Widmer, Julia Müller, Alissa Gübeli, Grzegorz Zuk, Osman Bilgin Gulcicek, Yuksel Altinel, Talar Vartanoglu, Emin Kose, Servet Rustu Karahan, Mehmet Can Aydin, Nuri Alper Sahbaz, Ilkay Halicioglu, Halil Alis, Ipek Sapci, Can Adıyaman, Ahmet Murat Pektaş, Turgut Bora Cengiz, Ilkan Tansoker, Vedatcan Işler, Muazzez Cevik, Deniz Mutlu, Volkan Ozben, Berk Baris Ozmen, Sefa Bayram, Sinem Yolcu, Berna Buse Kobal, Ömer Faruk Toto, Haluk Cem Çakaloğlu, Kagan Karabulut, Vahit Mutlu, Bahar Busra Ozkan, Saban Celik, Anil Semiz, Selim Bodur, Enisburak Gül, Busra Murutoglu, Reyyan Yildirim, Bahadir Emre Baki, Ekin Arslan, Ali Guner, Kadir Tomas, Nathan Walker, Nikhita Shrimanker, Michael Stoddart, Simon Cole, Ryan Breslin, Ravi Srinivasan, Mohamed Elshaer, Kristina Hunter, Ahmed Al‐Bahrani, Ignatius Liew, Nora Grace Mairs, Alistair Rocke, Lachlan Dick, Mobeen Qureshi, Debkumar Chowdhury, Naomi Wright, Clare Skerritt, Dorothy Kufeji, Adrienne Ho, Tharindra Dissanayake, Athula Tennakoon, Wadah Ali, Shujing Jane Lim, Charlene Tan, Stephen O'Neill, Catrin Jones, Stephen Knight, Dima Nassif, Abhishek Sharma, Oliver Warren, Rebecca White, Aia Mehdi, Nathan Post, Eliana Kalakouti, Enkhbat Dashnyam, Frederick Stourton, Ioannis Mykoniatis, Chelise Currow, Francisca Wong, Ashish Gupta, Veeranna Shatkar, Joshua Luck, Suraj Kadiwar, Alexander Smedley, Rebecca Wakefield, Philip Herrod, James Blackwell, Jonathan Lund, Fraser Cohen, Ashwath Bandi, Stefano Giuliani, Giles Bond‐Smith, Theodore Pezas, Neda Farhangmehr, Tomas Urbonas, Miklos Perenyei, Philip Ireland, Natalie Blencowe, Kirk Bowling, David Bunting, Lydia Longstaff, Neil Smart, Kenneth Keogh, Hyunjin Jeon, Muhammad Rafaih Iqbal, Shivun Khosla, Anna Jeffery, James Perera, Ella Teasdale, Ahmad Aboelkassem Ibrahem, Tariq Alhammali, Yahya Salama, Rakan Kabariti, Shaun Oram, Thomas Kidd, Fraser Cullen, Christopher Owen, Michael Wilson, Seehui Chiu, Hannah Sarafilovic, Jennifer Ploski, Elizabeth Evans, Athar Abbas, Sylvia Kamya, Norzawani Ishak, Carly Bisset, Cedar Andress, Ye Ru Chin, Priya Patel, David Evans, Anna Jeffery, James Perera, Aidan Haslegrave, Adam Boggon, Kirsten Laurie, Katie Connor, Thomas Mann, Dmitri Nepogodiev, Anahita Mansuri, Rachel Davies, Ewen Griffiths, Aized Raza Shahbaz, Calvin Eng, Farhat Din, Ariadne L'Heveder, Esther H.G. Park, Ramanish Ravishankar, Kirsten McIntosh, Jih Dar Yau, Luke Chan, Susan McGarvie, Lingshan Tang, Hui Lim, Suhhuey Yap, Jay Park, Zhan Herr Ng, Shahrukh Mirza, Yun Lin Ang, Luke Walls, Ella Teasdale, Chloe Roy, Simon Paterson‐Brown, Julian Camilleri‐Brennan, Kenneth Mclean, Michelle S D'Souza, Savva Pronin, David Ewart Henshall, Eunice Zuling Ter, Dina Fouad, Ashish Minocha, William English, Catrin Morgan, Dominic Townsend, Laura Maciejec, Shareef Mahdi, Onyinye Akpenyi, Elisabeth Hall, Hanaan Caydiid, Zakaria Rob, Tom Abbott, Hew D Torrance, Gareth Irwin, Robin Johnston, Mohammed Akil Gani, Gianpiero Gravante, Shivanchan Rajmohan, Kiran Majid, Shiva Dindyal, Christopher Smith, Madanmohan Palliyil, Sanjay Patel, Luke Nicholson, Neil Harvey, Katie Baillie, Sam Shillito, Suzanne Kershaw, Rebecca Bamford, Peter Orton, Elke Reunis, Robert Tyler, Wai Cheong Soon, Guled M. Jama, Dharminder Dhillon, Khyati Patel, Shayanthan Nanthakumaran, Rachel Heard, Kar Yan Chen, Behrad Barmayehvar, Uttaran Datta, Sivesh K Kamarajah, Sharad Karandikar, Sobhana Iftekhar Tani, Eimear Monaghan, Philippa Donnelly, Michael Walker, Jehangirshaw Parakh, Sarah Blacker, Anil Kaul, Arjun Paramasivan, Sameh Farag, Ashrafun Nessa, Salwa Awadallah, Jieqi Lim, James Chean Khun Ng, Katherine Gash, Ravi P. Kiran, Alice Murray, Eric Etchill, Mohini Dasari, Juan Puyana, Nadeem Haddad, Martin Zielinski, Asad Choudhry, Celeste Caliman, Mieshia Beamon, Therese Duane, Ragavan Narayanan, Mamta Swaroop, Jonathan Myers, Rebecca Deal, Erik Schadde, Mark Hemmila, Lena Napolitano, Kathleen To, Alex Makupe, Joseph Musowoya, Mayaba Maimbo, Niels Van Der Naald, Dayson Kumwenda, Alex Reece‐Smith, Kars Otten, Anna Verbeek, Marloes Prins, Alibeth Andres Baquero Suarez, Ruben Balmaceda, Chelsea Deane, Emilio Dijan, Mahmoud Elfiky, Laura Koskenvuo, Aurore Thollot, Bernard Limoges, Carmen Capito, Challine Alexandre, Henri Kotobi, Julien Leroux, Julien Rod, Kalitha Pinnagoda, Nicolas Henric, Olivier Azzis, Olivier Rosello, Poddevin Francois, Sara Etienne, Philippe Buisson, Sophian Hmila, Joe‐Nat Clegg‐Lamptey, Osman Imoro, Owusu Emmanuel Abem, Paul Wondoh, Dimitrios Papageorgiou, Vasiliki Soulou, Sabrina Asturias, Lenin Peña, Basant Kumar, Donal B O'Connor, Alberto Realis Luc, Alfio Alessandro Russo, Andrea Ruzzenente, Antonio Taddei, Camilla Cona, Corrado Bottini, Giovanni Pascale, Giuseppe Rotunno, Leonardo Solaini, Marco Maria Pascale, Margherita Notarnicola, Mario Corbellino, Michele Sacco, Paolo Ubiali, Roberto Cautiero, Tommaso Bocchetti, Elena Muzio, Vania Guglielmo, Eugenio Morandi, Patrizio Mao, Emilia De Luca, Margherita Notarnicola, Farah Mahmoud Ali, Justas Žilinskas, Kestutis Strupas, Paulius Kondrotas, Robertas Baltrunas, Juozas Kutkevicius, Povilas Ignatavicius, Choy Ling Tan, Jia Yng Siaw, Sir Young Yam, Ling Wilson, Mohamed Rezal Abdul Aziz, John Bondin, Carmina Diaz Zorrilla, Anass Majbar, Danjuma Sale, Lawal Abdullahi, Olabisi Osagie, Omolara Faboya, Adedeji Fatuga, Agboola Taiwo, Emeka Nwabuoku, Marte Bliksøen, Zain Ali Khan, Jazmin Coronel, Cesar Miranda, Idelso Vasquez, Luis M. Helguero‐Santin, Jennifer Rickard, Aurel Mironescu, Adesina Adedeji, Saleh Alqahtani, Max Rath, Michael Van Niekerk, Modise Zacharia Koto, Roel Matos‐Puig, Leif Israelsson, Tobias Schuetz, Mahmut Arif Yuksek, Meric Mericliler, Mehmet Uluşahin, Bernhard Wolf, Cameron Fairfield, Guo Liang Yong, Katharine Whitehurst, Michael Wilson, Natalie Redgrave, Caroluce K Musyoka, James Olivier, Kathryn Lee, Michael Cox, Muhamed M H Farhan‐Alanie, Rory Callan, Chali Chibuye, Tebian Hassanein Ahmed Ali, Syrine Rekhis, Muna Rommaneh, Oday Halhouli, Zi Hao Sam, Lawani Ismaïl, Vasileios Kalles, Francesco Pata, Gabriela Elisa Nita, Federico Coccolini, Luca Ansaloni, Thays Brunelli Pugliesi, Gabriel Pardo, Ruth Blanco

## Abstract

**Background:**

The World Health Organization (WHO) Surgical Safety Checklist has fostered safe practice for 10 years, yet its place in emergency surgery has not been assessed on a global scale. The aim of this study was to evaluate reported checklist use in emergency settings and examine the relationship with perioperative mortality in patients who had emergency laparotomy.

**Methods:**

In two multinational cohort studies, adults undergoing emergency laparotomy were compared with those having elective gastrointestinal surgery. Relationships between reported checklist use and mortality were determined using multivariable logistic regression and bootstrapped simulation.

**Results:**

Of 12 296 patients included from 76 countries, 4843 underwent emergency laparotomy. After adjusting for patient and disease factors, checklist use before emergency laparotomy was more common in countries with a high Human Development Index (HDI) (2455 of 2741, 89·6 per cent) compared with that in countries with a middle (753 of 1242, 60·6 per cent; odds ratio (OR) 0·17, 95 per cent c.i. 0·14 to 0·21, *P* < 0·001) or low (363 of 860, 42·2 per cent; OR 0·08, 0·07 to 0·10, *P* < 0·001) HDI. Checklist use was less common in elective surgery than for emergency laparotomy in high‐HDI countries (risk difference −9·4 (95 per cent c.i. −11·9 to −6·9) per cent; *P* < 0·001), but the relationship was reversed in low‐HDI countries (+12·1 (+7·0 to +17·3) per cent; *P* < 0·001). In multivariable models, checklist use was associated with a lower 30‐day perioperative mortality (OR 0·60, 0·50 to 0·73; *P* < 0·001). The greatest absolute benefit was seen for emergency surgery in low‐ and middle‐HDI countries.

**Conclusion:**

Checklist use in emergency laparotomy was associated with a significantly lower perioperative mortality rate. Checklist use in low‐HDI countries was half that in high‐HDI countries.

## Introduction

The volume of surgical procedures performed worldwide is large^1^ and, although many advances have been made in the past several decades, surgical care exposes patients to substantial risk of morbidity and mortality. The safety of surgical care has gained traction within the global health landscape, yet it remains a pressing concern in both resource‐rich and ‐poor settings^2^. In 2009, the WHO released a 19‐item surgical safety checklist for implementation in countries around the world^3^. The checklist was designed to promote understanding and cohesive communication, and to ensure good practice among all surgical team members at three specific intervals: before induction of anaesthesia, before skin incision, and before the patient leaves the operating theatre^3^. Although the improvement in outcomes was dramatic^4^, uptake of this safety tool remains to be quantified on a large scale. Attention surrounding use of the checklist in resource‐limited settings is of particular relevance to surgical care and outcomes^4^.

Process‐related discrepancies, such as lack of a particular safe practice protocol, are chief contributors to adverse surgical events^5^. Despite the potential benefits of checklist use, there are numerous barriers to implementation^6^, ^7^. Dynamic educational and social factors, such as ambiguity and confusion around the purpose of the checklist and negative attitudes to checklist adoption among some team members, contribute to poor checklist uptake^8^, ^9^. In addition, in settings where resources are limited, completion of a checklist that focuses on unavailable items can seem pointless, for instance in the absence of pulse oximetry or antibiotics^10^, ^11^. Despite these barriers to implementation and completion, it has been suggested^5^ that absence of the checklist itself may serve as a major contributor to adverse surgical events.

Although data supporting the effectiveness of the checklist in fostering improved surgical outcomes are encouraging, studies in globally representative populations are uncommon. Furthermore, checklist outcomes have been studied largely within elective general surgery and subspecialty settings, with only a few studies examining checklist use in emergency care^12^, ^13^. Attitudes to checklist use by providers working in emergency settings can be negative^12^. A survey of obstetric care providers found that one‐third believed a checklist would be an inconvenience in emergencies^14^. Despite this, the benefit of checklist use does extend to emergency surgical care, as shown in an analysis of the original WHO checklist study in urgent operations across eight countries^13^. Importantly, it is also possible that the benefit of the checklist may be greatest in emergency situations, given the increased risks^15^.

Using a large, validated, global data set, this study aimed to compare reported use of the WHO Surgical Safety Checklist in patients undergoing emergency laparotomy and elective gastrointestinal surgery. Associations were sought between checklist use and perioperative mortality, accounting for country developmental level as well as patient and disease factors.

## Methods

### Study design and participants

Data were collected prospectively within two international, multicentre, observational cohort studies: GlobalSurg 1^16^ and GlobalSurg 2^17^. Both studies were performed by the GlobalSurg Collaborative group using prespecified published protocols (NCT02179112^18^, NCT02662231^19^). This collaborative methodology has been described elsewhere^20^. A UK National Health Service Research Ethics review considered both GlobalSurg 1 and GlobalSurg 2 exempt from formal research registration (South East Scotland Research Ethics Service, references NR/1404AB12 and NR/1510AB5). Individual centres obtained their own audit, ethical or institutional approval. Details of data validation have been described and published previously^16^, ^17^. Results of this analysis are reported according to Strengthening the Reporting of Observational Studies in Epidemiology (STROBE) guidelines^21^.

In both contributing studies, investigators from healthcare facilities worldwide that fulfilled the inclusion criteria were invited to participate and submit data using an online platform^22^. Small teams of investigators were recruited via email, social media and through personal contacts. Investigators collected data during at least one 2‐week interval during study windows. In GlobalSurg 1, investigators identified consecutive patients between 1 July 2014 and 21 December 2014^16^. This study included any patient undergoing emergency intraperitoneal surgery, defined in the study methods^16^. In GlobalSurg 2, investigators included consecutive patients between 4 January 2016 and 31 July 2016^17^. This study included all patients undergoing elective or emergency gastrointestinal surgery^17^. In both studies, patients were followed to day 30 after surgery, or for the duration of their inpatient stay in locations where follow‐up was not feasible. Local investigators uploaded records to a secure online database, using the Research Electronic Data Capture (REDCap) system^23^.

Data from both GlobalSurg 1 and GlobalSurg 2 were combined to create a multicentre data set. Variables were cross‐referenced and streamlined for coding consistency. Patients were then selected as having undergone emergency laparotomy or elective gastrointestinal surgery. Emergency laparotomy was captured using the definition from the UK National Emergency Laparotomy Audit^24^, adapted for global settings. Trauma laparotomy was included, whereas laparoscopic procedures and individuals aged less than 18 years were not included in the analysis.

### Variables

The primary outcome measure was 30‐day perioperative mortality, expressed as a proportion. Perioperative mortality was defined as ‘any death, regardless of cause, occurring within 30 days of surgery in or out of the hospital’^25^. The metric was calculated by dividing the number of perioperative deaths by the total number of included operations performed^26^. The primary explanatory variable was reported use of the WHO Surgical Safety Checklist. Checklist use was recorded as ‘no, not available’, ‘no, but available’, ‘yes’, or ‘unknown’ for each patient in the study. Reported use of the checklist was calculated as a proportion recorded as ‘yes’ of the total number of patients included. Countries were stratified into three tertiles according to the Human Development Index (HDI) rank^27^. This is a composite statistic of life expectancy, education and income indices published by the United Nations. HDI was chosen over purely economic measures of country development on the principle that ‘people and their capabilities should be the ultimate criteria for assessing the development of a country, not economic growth alone’^27^.

Patient‐level variables included age, sex, diabetes, smoking status and ASA fitness grade. For simplification, ASA was grouped as a score of less than III and one of III or more. Disease‐level variables included six major diagnostic groups: abdominal wall, benign foregut, benign midgut/hindgut, malignancy, trauma/injury and other. Operative characteristics, including the requirement for a bowel resection and the level of contamination, were also included.

### Power considerations

GlobalSurg 1 and GlobalSurg 2 both included *a priori* sample size calculations that accounted for the uncertainty in the data coming from collaborating countries^18^, ^19^. In making between‐group comparisons of checklist use by HDI country or urgency, a difference from 10 to 5 per cent can be shown with α = 5 per cent and β = 10 per cent (90 per cent power) with group sizes of 582.

### Statistical analysis

Differences between HDI tertiles were tested with Pearson's χ^2^ test and Kruskal–Wallis test for categorical and continuous variables respectively. Multivariable logistic regression models were used to adjust for confounding in analyses of checklist use and 30‐day perioperative mortality. Coefficients are expressed as odds ratios (ORs) with 95 per cent confidence intervals and *P* values derived from percentiles of 10 000 bootstrap replications. Models were constructed using the following principles: variables associated with outcome measures in previous studies were accounted for; demographic variables were included in model exploration; all first‐order interactions were checked and included in final models if found to be influential; final model selection was informed using a criterion‐based approach minimizing the Akaike information criterion and discrimination determined using the c‐statistic (area under the receiver operating characteristic (ROC) curve). Hierarchical models accounting for clustering within countries were not used in this analysis owing to colinearity between explanatory variables of interest and country. Model residuals were checked (residuals *versus* fitted values; normality plot of standardized deviance residuals; and residuals *versus* leverage), and goodness of model fit determined using the Hosmer–Lemeshow test.

To help translate model outputs to real‐life quantities of interest, bootstrapped simulations of model predictions were performed at specified co‐variable levels. This enables the results of regression analyses to be expressed as probabilities, with the intention of these being more intuitive to interpret than ORs.

All analyses were undertaken using the R Foundation statistical program R 3.4.14, with finalfit^28^ and dplyr packages (R Project for Statistical Computing, Vienna, Austria).

### Data sharing

The data set can be explored using an online visualization application at http://data.globalsurg.org.

## Results

**Figure 1:**
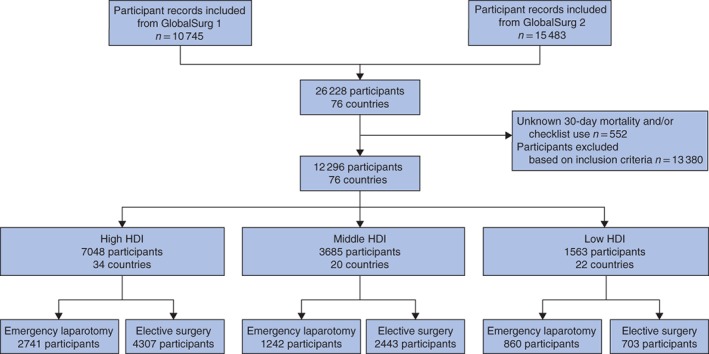
Flow chart of study population. HDI, Human Development Index

A total of 26 228 patient records were sourced from the GlobalSurg 1 (10 745, 41·0 per cent) and GlobalSurg 2 (15 483, 59·0 per cent) data sets (*Fig*. 1). For 552 patients (2·1 per cent) 30‐day mortality and/or checklist use was not known; these were removed. Patterns of missing data were examined and not considered to influence final results (*Tables S1* and *S2*, supporting information). For 13 380 patients (51·0 per cent) the inclusion criteria were not fulfilled, and these were removed; reasons included undergoing an emergency procedure other than emergency laparotomy and age less than 18 years. The included procedures by country HDI are shown in *Table S3* (supporting information). The final data set represents 12 296 patients from 76 countries, with 326 centres from GlobalSurg 1^16^ and 356 centres from GlobalSurg 2^17^.

### Demographics

Extensive data relating to patient and operative characteristics by HDI group and surgery type are provided in *Tables S4–S6* (supporting information) to allow a full understanding of the population. Patients were distributed across HDI groups as shown in *Fig*. 1. Patients undergoing emergency laparotomy were older, more likely to be men, had a higher ASA score, were less likely to have cancer, and had a higher rate of wound contamination (*Table S4*, supporting information). Those undergoing emergency laparotomy in low‐HDI compared with high‐HDI countries were younger, more likely to be men, had a lower ASA score, were less likely to have had a bowel resection or cancer, and more likely to have wound contamination (*Table S5*, supporting information). Similar differences were seen in the elective surgery group (*Table S6*, supporting information).

### Use of WHO Surgical Safety Checklist

Reported WHO Surgical Safety Checklist use across the entire cohort was 8881 of 12 296 (72·2 per cent). There was little difference in checklist use overall when comparing emergency laparotomy with elective surgery (73·7 *versus* 71·2 per cent respectively). Checklist use differed in high‐ (84·5 per cent) compared with middle‐ (59·4 per cent) and low‐ (47·3 per cent) HDI country groups (*Table* 1).

**Table 1:** Patient and operative characteristics by reported WHO Surgical Safety Checklist use

	Checklist used			
	No	Yes	Total	*P* †
Urgency				0·003
Emergency laparotomy	1272 (26·3)	3571 (73·7)	4843	
Elective surgery	2143 (28·8)	5310 (71·2)	7453	
HDI tertile				< 0·001
High	1095 (15·5)	5953 (84·5)	7048	
Middle	1496 (40·6)	2189 (59·4)	3685	
Low	824 (52·7)	739 (47·3)	1563	
Age (years)*	47·7(19·2)	53·8(19·7)	–	< 0·001‡
Sex				0·729
M	1612 (28·2)	4095 (71·8)	5707	
F	1682 (28·0)	4334 (72·0)	6016	
Missing	121 (21·1)	452 (78·9)	573	
ASA fitness grade				< 0·001
< III	2598 (30·2)	6006 (69·8)	8604	
≥ III	743 (21·2)	2766 (78·8)	3509	
Missing	74 (40·4)	109 (59·6)	183	
Smoker				0·009
No	2594 (29·2)	6302 (70·8)	8896	
Yes	670 (26·5)	1858 (73·5)	2528	
Missing	151 (17·3)	721 (82·7)	872	
Diabetes				< 0·001
No	3076 (28·4)	7744 (71·6)	10 820	
Yes	339 (23·0)	1137 (77·0)	1476	
Disease classification				< 0·001
Abdominal wall	174 (29·8)	409 (70·2)	583	
Other	1567 (32·2)	3305 (67·8)	4872	
Benign foregut	478 (27·9)	1235 (72·1)	1713	
Benign midgut/hindgut	410 (20·8)	1558 (79·2)	1968	
Malignancy	569 (22·2)	1994 (77·8)	2563	
Trauma/injury	190 (37·3)	320 (62·7)	510	
Missing	27 (31)	60 (69)	87	
Bowel resection				< 0·001
No	1879 (25·7)	5435 (74·3)	7314	
Yes	1535 (30·8)	3442 (69·2)	4977	
Missing	1 (20)	4 (80)	5	
Malignancy				< 0·001
No	2846 (29·2)	6887 (70·8)	9733	
Yes	569 (22·2)	1994 (77·8)	2563	
Contamination				0·061
Clean/contaminated	2564 (27·3)	6840 (72·7)	9404	
Contaminated	324 (27·1)	873 (72·9)	1197	
Dirty	476 (30·1)	1106 (69·9)	1582	
Missing	51 (45·1)	62 (54·9)	113	

Values in parentheses are percentages unless indicated otherwise;

* values are mean(s.d.). HDI, Human Development Index.

† χ^2^ test, except

‡ Kruskal–Wallis test (comparisons of available data).

**Figure 2:**
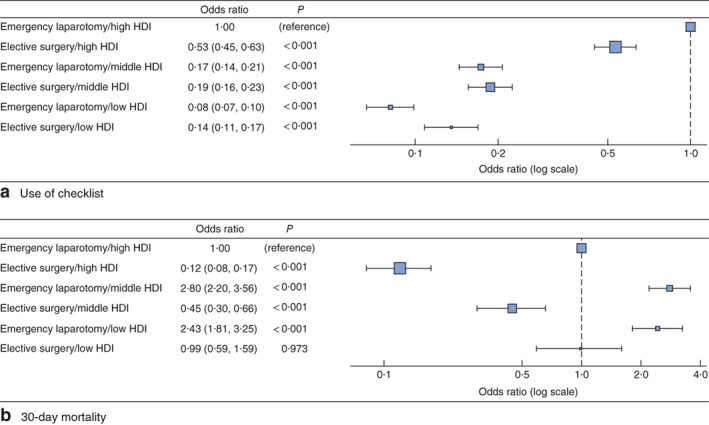
Odds ratio plots of WHO Surgical Safety Checklist use and 30‐day mortality. **a** Use of WHO checklist and **b** 30‐day mortality for surgery type and Human Development Index (HDI) group from multivariable logistic regression models. Odds ratios are shown with 95 per cent confidence intervals and *P* values. Checklist use was adjusted for age, ASA score, diabetes status, disease classification, bowel resection and wound contamination. For full models, see *Tables S7* and *S8* (supporting information). Mortality (**b**) is adjusted for WHO surgical safety checklist use, age, ASA, disease classification, bowel resection and wound contamination

A multivariable regression model was used to adjust for confounding and identify predictors of checklist use (*Table S7*, supporting information). A significant interaction was found between type of surgery and country HDI for checklist use: the patterns of checklist use by surgery type were different across HDI groups. After adjusting for patient and operative characteristics, checklist use continued to be lower in low‐ and middle‐HDI countries (*Fig*. 2 *a*). To convey differences in checklist use more intuitively, bootstrapped predicted probabilities of checklist use were determined (*Table* 2). Absolute risk differences for emergency laparotomy *versus* elective surgery differed by HDI group. Checklist use was less common for elective surgery than emergency laparotomy in the high‐HDI group (absolute risk difference −9·4 (95 per cent c.i. −11·9 to −6·9) per cent; *P* < 0·001), no different in the middle‐HDI group (1·9 (−2·3 to 6·5) per cent; *P* = 0·357) and more common in the low‐HDI group (12·1 (7·0 to 17·3) per cent; *P* < 0·001).

**Table 2:** WHO Surgical Safety Checklist reported use by country Human Development Index and type of surgery

	Checklist used*					
	No	Yes	*P*§	Adjusted probability of checklist use (%)†	Absolute risk difference (%)†	*P*
High HDI						
Emergency laparotomy	286 (10·4)	2455 (89·6)		86·0 (83·4, 88·3)	–	
Elective surgery	809 (18·8)	3498 (81·2)	< 0·001	76·6 (73·8, 79·3)	−9·4 (–11·9, −6·9)	< 0·001
Middle HDI						
Emergency laparotomy	489 (39·4)	753 (60·6)		51·6 (46·4, 56·7)	–	
Elective surgery	1007 (41·2)	1436 (58·8)	0·280	53·6 (49·4, 57·5)	1·9 (–2·3, 6·5)	0·357
Low HDI						
Emergency laparotomy	497 (57·8)	363 (42·2)		33·3 (28·4, 38·4)	–	
Elective surgery	327 (46·5)	376 (53·5)	< 0·001	45·4 (40·2, 50·9)	12·1 (7·0, 17·3)	< 0·001

Values in parentheses are *percentages and †95 per cent confidence intervals. A multivariable logistic regression model was specified for checklist use by Human Development Index (HDI) group, type of surgery and confounders (see *Fig*. 2 and *Table S4*, supporting information). Bootstrapped adjusted predictions of the probability of checklist use were performed for different HDI groups and surgery type, with other co‐variable levels specified: age 52 years; ASA grade less than III; no diabetes; malignancy disease classification; clean/contaminated wound status. Absolute risk differences for the probability of checklist use were determined and a two‐sided *P* value was calculated. §χ^2^ test (within HDI group between surgery type and checklist).

### Mortality in emergency laparotomy

Overall, 30‐day mortality after emergency laparotomy (621 of 4843, 12·8 per cent) was ten‐times higher than for elective surgery (94 of 7453, 1·3 per cent) (*Table S8*, supporting information). There was notable variation in mortality after elective surgery by HDI group, but less variation after emergency laparotomy in the unadjusted analysis. However, after adjustment for confounding, significant differences were seen in 30‐day mortality after emergency laparotomy in low‐ (OR 2·43, 95 per cent c.i. 1·81 to 3·25; *P* < 0·001) and middle‐ (2·80, 2·20 to 3·56; *P* < 0·001) HDI groups compared with the high‐HDI group (*Fig*. 2 *b*; *Table S8*, supporting information). Thirty‐day mortality after elective surgery in low‐HDI countries was equivalent to 30‐day mortality after emergency laparotomy in high‐HDI countries.

### Use of checklist and 30‐day mortality

**Figure 3:**
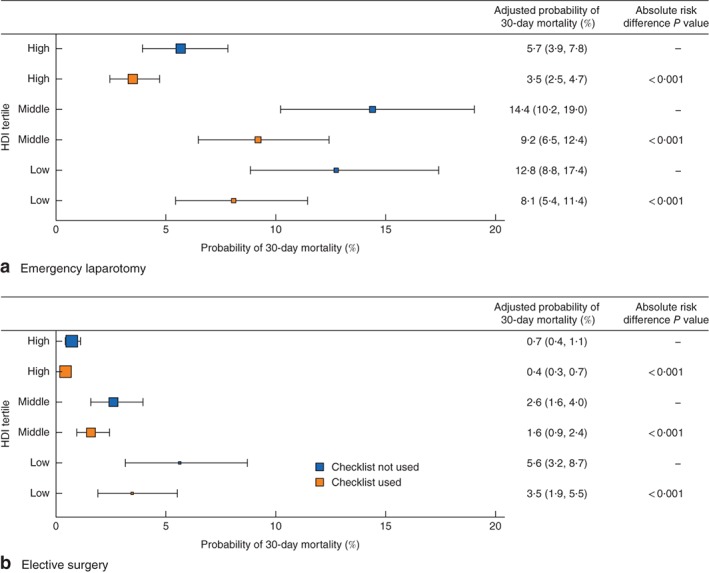
Adjusted probability of 30‐day mortality by surgery type, Human Development Index group and WHO Surgical Safety Checklist use. **a** Emergency laparotomy; **b** elective surgery. The multivariable logistic regression model for 30‐day mortality (*Fig*. 2; *Table S8*, supporting information) was used to generate adjusted predicted probabilities of death using bootstrap replication, with other co‐variable levels specified: age 52 years, ASA grade less than III, malignancy disease classification, and contamination. Absolute risk differences for 30‐day mortality are presented with 95 per cent confidence intervals, and two‐sided *P* values for the absolute risk difference (*Table S9*, supporting information). HDI, Human Development Index

Overall, reported use of the WHO Surgical Safety Checklist was associated with a lower 30‐day mortality (471 of 8881, 5·3 per cent) compared with reported non‐use (244 of 3415, 7·1 per cent) (*Table S8*, supporting information). In models adjusting for confounders, reported use of the checklist was still associated with a significantly lower 30‐day mortality (OR 0·60, 95 per cent c.i. 0·50 to 0·73; *P* < 0·001). Again, to create a more intuitive interpretation of the mortality model, adjusted predicted probabilities of 30‐day mortality were created to allow comparisons across HDI group, surgery type and reported checklist use (*Fig*. 3; *Table S9*, supporting information). No interaction between checklist use and 30‐day mortality was seen for HDI or type of surgery. Thus, as expected, a significant checklist effect was seen for each combination of surgery type and HDI group, with magnitudes of effect shown in *Fig*. 3. The greatest absolute risk difference for checklist use was seen in emergency surgery in low‐HDI (absolute risk reduction 4·6 (95 per cent c.i. 2·7 to 7·0) per cent; *P* < 0·001) and middle‐HDI (5·1 (2·9 to 7·8) per cent; *P* < 0·001) countries, by virtue of the higher baseline 30‐day mortality (*Table S9*, supporting information).

## Discussion

In this large multinational prospective cohort study, reported use of the WHO Surgical Safety Checklist was associated with a significant reduction in 30‐day perioperative mortality. This relationship was consistent and independent of key patient and disease‐related variables. Checklist use in low‐HDI countries was half that of high‐HDI countries, and this effect persisted after accounting for differences in patient and disease characteristics. Checklist use was lower for elective surgery than for emergency laparotomy in high‐HDI countries; this finding was unexpected. The association between checklist use and lower mortality was consistent across HDI groups and type of surgery, even after adjustment for case mix. The greatest absolute benefits were found in emergency surgery in low‐ and middle‐HDI countries, owing to the higher baseline mortality rate.

Evidence supporting use of the surgical safety checklist in hospital practice is widely positive and supports the promotion of the checklist in patient safety programmes worldwide^2^. A strength of this study is the breadth of countries and hospitals that contributed prospectively collected data. Use of the WHO Surgical Safety Checklist in low‐ and middle‐income countries was 2928 of 5248 (55·8 per cent), the same as that reported in the recent African Surgical Outcomes Study (6183 of 10 836, 57·1 per cent)^29^. In the present study, checklist use was found to be significantly lower in some low‐ and middle‐HDI countries compared with high‐HDI countries, yet the association with lower mortality was still seen. This clearly highlights an area for practice change. Fostering awareness to motivate local checklist champions is important, but may be difficult in more remote environments. In regions with less established local organizational infrastructure, the checklist may simply have not come to the attention of providers. Supportive governmental and academic institutions are crucial in facilitating the process, through continued professional development and ministry of health‐accredited programmes^30^. Successful implementation of the checklist requires careful thought and local adaptation. Avoiding the perception of the checklist as just a ‘tickbox’ exercise is crucial for success^9^.

This study explored the hypothesis that checklist use is lower in emergency settings due to the particular challenges therein. Focus on emergency laparotomy provided a more homogeneous study group, rather than including all patients undergoing emergency surgery. Checklist use has been studied extensively in elective surgery, but has received less attention in emergency surgery, particularly in global settings^13^. Narratives that highlight time pressures, low staffing, inflexible hierarchies and lack of resources during times of emergency promote an idea that the checklist should not be prioritized in urgent care. The present study shows that the checklist can be used in emergency settings, and commonly is. Moreover, associations between checklist use and better outcomes are just as evident in emergency laparotomy as in elective surgery. The capacity for checklist implementation in settings providing emergency surgical care should not be undervalued. On the contrary, there may be much to gain for emergency surgery in low‐ and middle‐income settings, given the higher baseline mortality rate.

A number of weaknesses to the approach taken here may be discussed. In general, the methodology is subject to selection bias at hospital level. Collaborators self‐select to take part, which may reflect better resourced institutions contributing than is seen in the general population. During the study period, consecutive patients must be recruited so that selection bias at patient level is minimized. Data were validated particularly carefully in the GlobalSurg 2 study^17^. As described previously, patient recruitment together with a subset of variables were re‐collected by an independent team in contributing hospitals. In centres with the fewest resources, this can be a challenge, particularly where there are no formal written records. Some collaborators described the GlobalSurg data as of better quality than what was otherwise available within their hospital.

With regard to checklist use itself, collaborators simply reported whether a WHO Surgical Safety Checklist had been used before surgery. No review was undertaken of what form the checklist took, what local adaptations may have been introduced, whether there was broad team acceptance of the process, or whether the implementation was deemed successful. Self‐reported checklist use is a poor reflection of meaningful compliance with checklist items^31^, and partial compliance reduces the positive benefits on outcomes derived from this^32^. Furthermore, the use and appropriate implementation of a surgical safety checklist may represent a wide range of health service system characteristics, including organizational and management attributes that support good clinical practices. Facilities reporting high use may also be those with significant resources, staffed by individuals familiar with key patient safety concepts who work together to ensure reliable systems are in place to deliver consistent patient care.

This study shows that the WHO Surgical Safety Checklist can be used in emergency surgery in resource‐poor settings. The association with lower mortality is likely to reflect broader health system differences that prioritize safe and effective surgical care, yet the checklist plays an important part. Much of the benefit is likely to come from behaviours that can be difficult to measure, such as improved communication, better team work, identification of potential problems before they occur, and empowerment of members of staff at all levels. The checklist likely helps improve surgical safety by providing a framework for focusing teams on specific critical safety standards that are frequently assumed to have occurred but may not be adhered to. It can also help identify specific lapses and process weaknesses that can be the focus of improvement efforts. Where standards are either not known or not clear, the checklist can raise awareness of them and help guide hospital policies and protocols. It can even create a ‘team‐generated Hawthorne effect’, whereby all perioperative personnel are involved in the responsibility of ensuring compliance with standards, and observe completion together using the checklist.

The data reported here have important implications for policy‐makers. Ten years after the introduction of the checklist, there is much work to be done in promoting its adoption worldwide. Local adaptation and ownership are clearly important in ensuring long‐term sustainable change^33^. Further studies around the details of implementation in resource‐constrained settings will help tailor checklist procedures to local needs, thereby ensuring greatest effect. Strong compliance and effective implementation are challenging, but have the potential to save many lives and should be a priority for surgical safety.

## Supporting information


**Table S1 Pattern of missing data for 30‐day mortality by other patient and operative characteristics**. Data are n (%) unless otherwise stated. P‐values are for comparisons across non‐missing data and are chi‐squared or Kruskal‐Wallis (*) tests. HDI, human development index; ASA, American Society of Anesthesiologists Physical Classification System; SD, standard deviation.
**Table S2 Pattern of missing data for reported WHO Surgical Safety Checklist use by other patient and operative characteristics**. Data are n (%) unless otherwise stated. P‐values are for comparisons across non‐missing data and are chi‐squared or Kruskal‐Wallis (*) tests. HDI, human development index; ASA, American Society of Anesthesiologists Physical Classification System; SD, standard deviation.
**Table S3 Procedures by human development index group**
**Table S4 Patient and operative characteristics by type of surgery**. Data are n (%) unless otherwise stated. P‐values are for comparisons across non‐missing data and are chi‐squared or Kruskal‐Wallis (*) tests. HDI, human development index; ASA, American Society of Anesthesiologists Physical Classification System; SD, standard deviation.
**Table S5 Patient and operative characteristics by country human development index for emergency laparotomy**. Data are n (%) unless otherwise stated. P‐values are for comparisons across non‐missing data and are chi‐squared or Kruskal‐Wallis (*) tests. HDI, human development index; ASA, American Society of Anesthesiologists Physical Classification System; SD, standard deviation.
**Table S6 Patient and operative characteristics by country human development index for elective surgery**. Data are n (%) unless otherwise stated. P‐values are for comparisons across non‐missing data and are chi‐squared or Kruskal‐Wallis (*) tests. HDI, human development index; ASA, American Society of Anesthesiologists Physical Classification System; SD, standard deviation.
**Table S7 Univariable and multivariable logistic regression analysis of reported WHO Surgical Safety Checklist use**. Data are n (%) unless otherwise stated. HDI, human development index; ASA, American Society of Anesthesiologists Physical Classification System; SD, standard deviation. Number in data frame = 12296, Number in model = 11911, Missing = 385, AIC = 12598·8, C‐statistic = 0·717, H&L = Chi‐sq(8) 67·60 (p<0·001).
**Table S8 Univariable and multivariable logistic regression analysis of 30‐day mortality**. Data are n (%) unless otherwise stated. HDI, human development index; ASA, American Society of Anesthesiologists Physical Classification System; SD, standard deviation. Number in data frame = 12296, Number in model = 11916, Missing = 380, AIC = 3947·7, C‐statistic = 0·87, H&L = Chi‐sq(8) 14·28 (p=0·075).
**Table S9 Probability of 30‐day mortality by surgery type, human development index group and WHO Surgical Safety Checklist use**. The multivariable logistic regression model for 30‐day mortality (Figure 2 and supplementary table 5) was used to generate adjusted predicted probabilities of death using bootstrap replication.
**Appendix S1 Members of the GlobalSurg Collaborative**
